# Initial high-resolution microscopic mapping of active and inactive regulatory sequences proves non-random 3D arrangements in chromatin domain clusters

**DOI:** 10.1186/s13072-017-0146-0

**Published:** 2017-08-07

**Authors:** Marion Cremer, Volker J. Schmid, Felix Kraus, Yolanda Markaki, Ines Hellmann, Andreas Maiser, Heinrich Leonhardt, Sam John, John Stamatoyannopoulos, Thomas Cremer

**Affiliations:** 1 LMU Biocenter, Department Biology II, Ludwig Maximilians-Universität (LMU Munich), Grosshadernerstr. 2, 82152 Martinsried, Germany; 2 BioImaging Group, Department of Statistics, Ludwig Maximilians-Universität (LMU Munich), Munich, Germany; 3 Department of Biochemistry and Molecular Biology, Monash Biomedicine Discovery Institute, Monash University, Melbourne, 3800 Australia; 4 Department of Biological Chemistry, David Geffen School of Medicine at UCLA, Los Angeles, CA USA; 5 Department of Genome Sciences, University of Washington, Seattle, WA USA; 6 Center for Cancer Research, National Cancer Institute, Bethesda, MD USA

**Keywords:** Transcription regulatory sequences, DNAse I hypersensitive sites, Super-resolution microscopy, Chromatin domain, Nuclear architecture, Active and inactive nuclear compartment, Chromatin compaction

## Abstract

**Background:**

The association of active transcription regulatory elements (TREs) with DNAse I hypersensitivity (DHS[+]) and an ‘open’ local chromatin configuration has long been known. However, the 3D topography of TREs within the nuclear landscape of individual cells in relation to their active or inactive status has remained elusive. Here, we explored the 3D nuclear topography of active and inactive TREs in the context of a recently proposed model for a functionally defined nuclear architecture, where an active and an inactive nuclear compartment (ANC–INC) form two spatially co-aligned and functionally interacting networks.

**Results:**

Using 3D structured illumination microscopy, we performed 3D FISH with differently labeled DNA probe sets targeting either sites with DHS[+], apparently active TREs, or DHS[−] sites harboring inactive TREs. Using an in-house image analysis tool, DNA targets were quantitatively mapped on chromatin compaction shaped 3D nuclear landscapes. Our analyses present evidence for a radial 3D organization of chromatin domain clusters (CDCs) with layers of increasing chromatin compaction from the periphery to the CDC core. Segments harboring active TREs are significantly enriched at the decondensed periphery of CDCs with loops penetrating into interchromatin compartment channels, constituting the ANC. In contrast, segments lacking active TREs (DHS[−]) are enriched toward the compacted interior of CDCs (INC).

**Conclusions:**

Our results add further evidence in support of the ANC–INC network model. The different 3D topographies of DHS[+] and DHS[−] sites suggest positional changes of TREs between the ANC and INC depending on their functional state, which might provide additional protection against an inappropriate activation. Our finding of a structural organization of CDCs based on radially arranged layers of different chromatin compaction levels indicates a complex higher-order chromatin organization beyond a dichotomic classification of chromatin into an ‘open,’ active and ‘closed,’ inactive state.

**Electronic supplementary material:**

The online version of this article (doi:10.1186/s13072-017-0146-0) contains supplementary material, which is available to authorized users.

## Background

Transcription regulatory elements (TREs) such as promoters, enhancers or insulators comprise non-coding sequences located within or in close vicinity to a gene but are also found up to ~1 Mb distally from their genes [1, 2]. Active TREs are characterized by a local ‘open’ chromatin conformation [3–5] associated with nucleosome displacement and specific histone signatures [6, 7] and were shown to have an increased sensitivity to DNAse I digestion, constituting DNAse I hypersensitive sites (DHS[+] of ~100–1000 bp [8–11]. Genome-wide profiling in various human cell types averaged over large cell populations identified >10,000 DHS[+] clusters. Approximately 10% were found to be cell type specific [2, 10, 12]. These sites encompass all experimentally validated cis-regulatory sequences; thus, DHS[+] clusters typically signify the location of active TREs in a genome.

The spatial organization of TREs has been addressed in terms of their contact frequencies with defined chromatin segments by chromosome conformation capture (Hi-C) or ChIP-Seq analyses [13–17], where genome-wide detection of DHS[+] sites in single cells was recently achieved by an ultrasensitive DNase sequencing strategy [18]. Still, little is known about the global 3D and 4D organization of regulatory sequences within the nuclear landscape of individual cells. Accordingly, it is unknown to which extent ‘open’ and ‘closed’ chromatin configurations may guide and constrain the accessibility of transcription factors (TFs) or chromatin modifiers to regulatory sequences. This issue has gained interest in the context of strong evidence for a distinct, but also dynamic nuclear architecture [19–24].

Microscopic investigations have demonstrated a structural organization of chromosome territories (CTs) built up from ~1-Mb chromatin domains (CDs) [25–27]. In particular, fluorescence labeling of specific genomic regions combined with 3D super-resolved microscopy has provided unprecedented opportunities to study nuclear arrangements of specific chromatin structures at the single-cell level and their cell-to-cell variability [28–31]. These studies have indicated that ~1-Mb CDs are composed of smaller subdomains (subCDs) and also form larger chromatin domain clusters (CDCs) [21, 26]. Genome-wide chromosome conformation capture methods have confirmed the territorial organization of chromosomes in mammalian cell nuclei and led to the discovery of ~1-Mb-sized topologically associating domains (TADs) [32–35]. TADs are built from smaller subdomains [32, 36] but also form larger units, called ‘metaTADs’ [37]. CDs and TADs reflect higher-order chromatin entities [38, 39], which provide the structural backbone for tissue-specific regulatory interactions [23, 40, 41].

In our present study, we explored the feasibility of 3D-FISH, 3D structured illumination microscopy (3D-SIM) and new 3D image analysis tools to determine the 3D nuclear topography of active and inactive TREs defined by their DHS[+] or DHS[−] status in human fibroblasts (BJ1 cells) and an adenocarcinoma cell line (A549). Below we use the terms DHS[+] interchangeably for sites with active TREs and DHS[−] for sites lacking (active) TREs. 3D FISH experiments were performed with an appropriately adapted protocol, which was previously shown to preserve key characteristics of the nuclear ultrastructure discernible at the resolution level of 3D-SIM [30, 42, 43], which is set at ~120 nm lateral and 250–300 nm axial [44, 45]. Highly resolved, quantitative measurements of DAPI intensities after DNA staining of nuclei were used as a proxy for local differences in chromatin compaction [46]. We describe the 3D topography of regulatory sequences in the context of a recently proposed model for a functionally defined nuclear landscape, where an active and an inactive nuclear compartment (termed ANC and INC) form two spatially co-aligned and functionally interacting 3D networks (for review, see [21] and Additional file 1 for illustration). The INC is formed by compact chromatin domains with low transcriptional activity for coding genes, which forms the interior core of a chromatin domain cluster (CDC). This compact core of CDCs is lined by a peripheral layer of decondensed, transcriptionally active chromatin domains, enriched in marks for transcriptionally competent chromatin termed perichromatin region (PR) [43, 47, 48]. The PR lines a contiguous channel system, the interchromatin compartment (IC), which starts at nuclear pores and permeates between CDs/CDCs [43, 48]. In addition to its potential role in nuclear import/export functions, the IC harbors nuclear bodies required for functions occurring within the PR. Accordingly, IC and PR together are considered as the active nuclear compartment (ANC).

According to the ANC–INC network model, we expected an enrichment of active TREs within the ANC, whereas a location of inactive TREs seemed possible within either the ANC or the INC. In case of inactive TREs embedded within the INC, their activation would correlate with a relocation toward the ANC. Our study argues for a non-random distinct distribution of targeted sites: Segments harboring active TREs are typically exposed at the outer periphery of CDCs constituting the active nuclear compartment (ANC), whereas inactive TREs are enriched toward the more compacted interior of CDCs, constituting the INC.

## Results

### Semi-automated quantitative 3D mapping of DNA sequences on chromatin compaction-defined nuclear landscapes

**Fig. 1:**
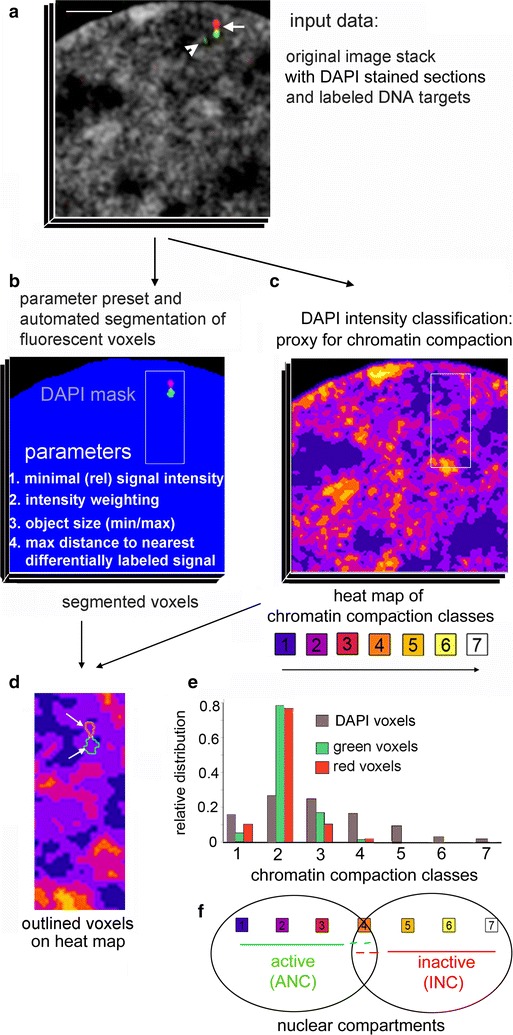
Workflow for quantitative mapping of specific FISH signals on 3D chromatin compaction maps. **a** Representative part of a section from an original 3D-SIM image stack of a whole nucleus acquisition shown by the example of a BJ1 nucleus. Chromatin counterstained with DAPI (*gray*), two adjacent DNA targets visualized by *green* and *red* fluorescent signals (*arrow*). The *arrowhead points* to an additional small *green* fluorescent (background) signal. *Scale bar* 2 µm. **b** Segmented fluorescent voxels of FISH signals within DAPI mask after defined parameter settings. The small isolated *green signal* seen in (*arrowhead* in **a**) is discarded due to a distance >0.5 µm from the nearest *red signal* centroid, set as limit between two differently labeled targets. **c** Same section after classification of DAPI signals into seven intensity classes as proxy for chromatin compaction visualized as *color* heat map. **d** *Inset* magnification from framed area in **b** and **c** with *outlined green* and *red* segmented signals. **e** Relative distribution of *green* and *red* fluorescent voxels in this nucleus mapped on DAPI intensity-defined chromatin compaction classes (*gray*). **f** Assignment of the active and inactive nuclear compartment (ANC/INC) linked to chromatin compaction classes, for explanation see text

For our investigation, we developed a semi-automated approach for 3D mapping of FISH signals on chromatin compaction-defined nuclear landscapes [46] based on optical serial sections of DAPI-stained nuclei recorded with 3D-SIM. 3D mapping of specific hybridization signals is exemplified in Fig. 1a with two contiguous, differentially labeled 6-kb probes (see below for details and application of these probes). For an unbiased 3D assessment of probe signals, defined preset values such as minimal target size, relative signal intensity and a maximal distance between the centroid position of a given signal to its nearest differently labeled signal were applied for signal segmentation (Fig. 1b). An algorithm for chromatin compaction classification of DAPI-stained nuclei was employed to generate seven DAPI intensity classes with equal intensity variance as a measure for chromatin compaction [46]. Classes of chromatin compaction can be visualized as a color-coded heat map (Fig. 1c). Voxels assigned to class 1 (blue) depict regions with or close to background DAPI intensities, representing the largely DNA-free interchromatin compartment (IC), classes 2–3 (purple and deep red) comprise chromatin with low DAPI intensity, representing decondensed (‘open’) chromatin. Class 4 (dark orange) is considered as an intermediate zone, classes 5–7 (orange, yellow, white) comprise ‘closed’ chromatin. 3D coordinates of segmented FISH signals are spatially mapped on chromatin compaction classes (Fig. 1d) and plotted with their relative distributions on the respective classes. Figure 1e exemplifies a highly non-random distribution of 3D FISH targets within the seven chromatin compaction classes. Classes 1–3 represent the active nuclear compartment, class 4 an intermediate zone, classes 5–7 the INC (Fig. 1f, for review, see [21]).

### Multilayered shell-like organization of chromatin domain clusters

**Fig. 2:**
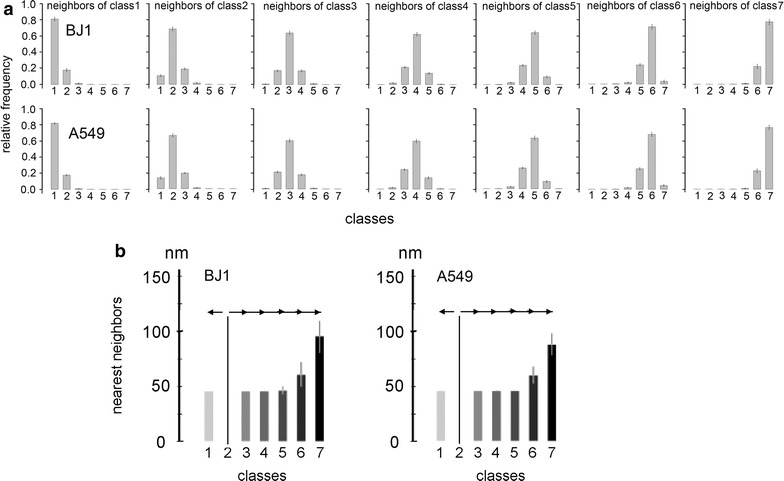
Quantitative assessment for a compartmentalized organization of distinct chromatin compaction classes. **a** Frequency of chromatin compaction class for each nearest-neighbor voxel of a given compaction class in BJ1 (*N* = 45) and A549 (*N* = 30). **b** Minimal average distances of each voxel of class 2 to the nearest voxel assigned to all other classes

Color heat maps of DAPI-stained nuclear SIM sections (as exemplified in Fig. 1c, d) suggest a multilayered shell-like chromatin organization of CDCs with compact chromatin (classes 5–7) typically located in their interior, surrounded by a decondensed peripheral layer (classes 2–3) lined by the IC (class 1). The visual impression of a radially arranged compartmentalization of chromatin layers is supported by quantitation of nearest-neighbor voxels of a given chromatin compaction class in 3D SIM serial sections of BJ1 (*N* = 45) and A549 (*N* = 30) cell nuclei (Fig. 2a). Most nearest neighbors belong to the same intensity class, a smaller fraction to the next higher or lower class and only rare voxels to remote classes. For a rough estimate of minimal distances required for potential movements of any target DNA from the most compact (interior) to the most decondensed (peripheral) part of CDCs, the distance for each voxel assigned to class 2 to the nearest voxel assigned to all other classes was measured. Minimal average distances indicate that movements of ~100 nm may suffice for a relocation of a sequence between the most inner and the most outer layer of CDCs (Fig. 2b).

### 3D topography of active and inactive TREs in chromatin domain clusters

**Fig. 3:**
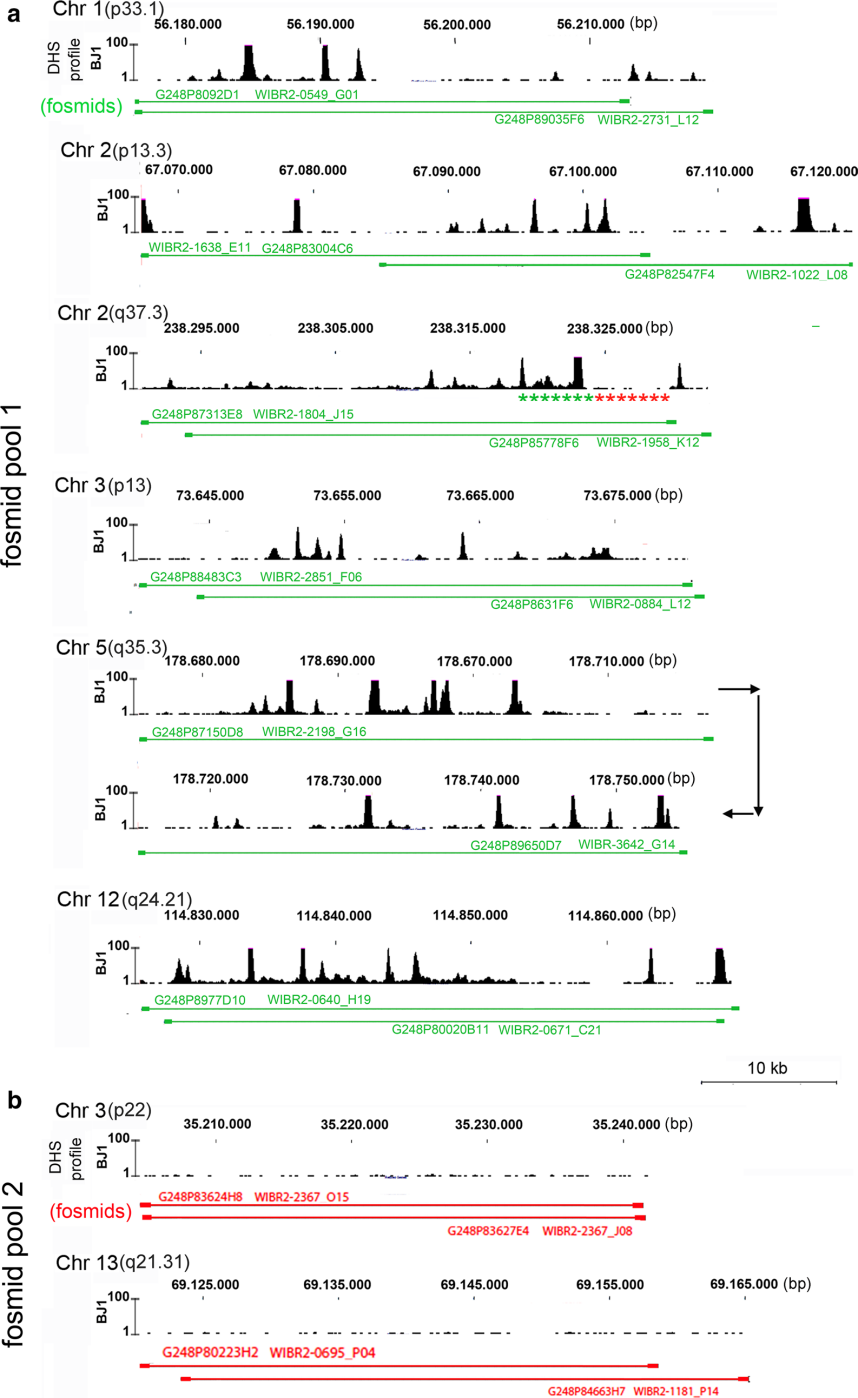
DHS profiles of targeted genomic regions representing DHS[+] or DHS[−] sites on different chromosomes in BJ1 nuclei and fosmid clones used for their delineation by 3D-FISH. **a** Selected regions with interspersed clusters of DHS[+] sites, DHS profiles shown in *black* (*browser shots* adopted from http://encodeproject.org/). Genomic position and assignment to DHS profiles of fosmid clones used in fosmid pool 1 are indicated by *green lines*. **b** Selected regions representing DHS[−] sites, DHS profiles shown in *black* (*browser shots* adopted from http://encodeproject.org/). Genomic position and assignment to DHS profiles of fosmid clones used in fosmid pool 2 are indicated by *red lines*. Using pairs of (partially) overlapping clones for both fosmid pools ensures optimal hybridization efficiency. *Asterisks* mark the approximate location of probes described in Fig. 5

For the coverage of DHS[+] and DHS[−] sites located on different chromosomes in BJ1 cells, we used two differently labeled DNA probe sets of pooled fosmid clones with each fosmid carrying a human sequence of ~40 kb (Fig. 3). DHS profiles of the respective sites were identified on availability of NIH Roadmap Epigenomics Mapping Consortium data (www.roadmapepigenomics.org hg19). Fosmid pool 1 comprises six genomic targets located on chromosome bands 1p33.1, 2p13.3, 2q37.3, 3p13, 5q35.3 and 12q24.21 peppered with numerous DHS[+] clusters expanding over several kb (Fig. 3a). These targets contain different types of active TREs (for type and ‘open’ chromatin marks, see Additional file 2). Fosmid pool 2 (Fig. 3b) comprises two DHS[−] segments on chromosome bands 3p22 and 13q21.31. Several TREs were identified in these segments providing evidence for regulatory potential in these regions [49, 50]. However, in line with their DHS[−] status none of these regions shows ‘open’ chromatin marks in fibroblasts (Additional file 2).

**Fig. 4:**
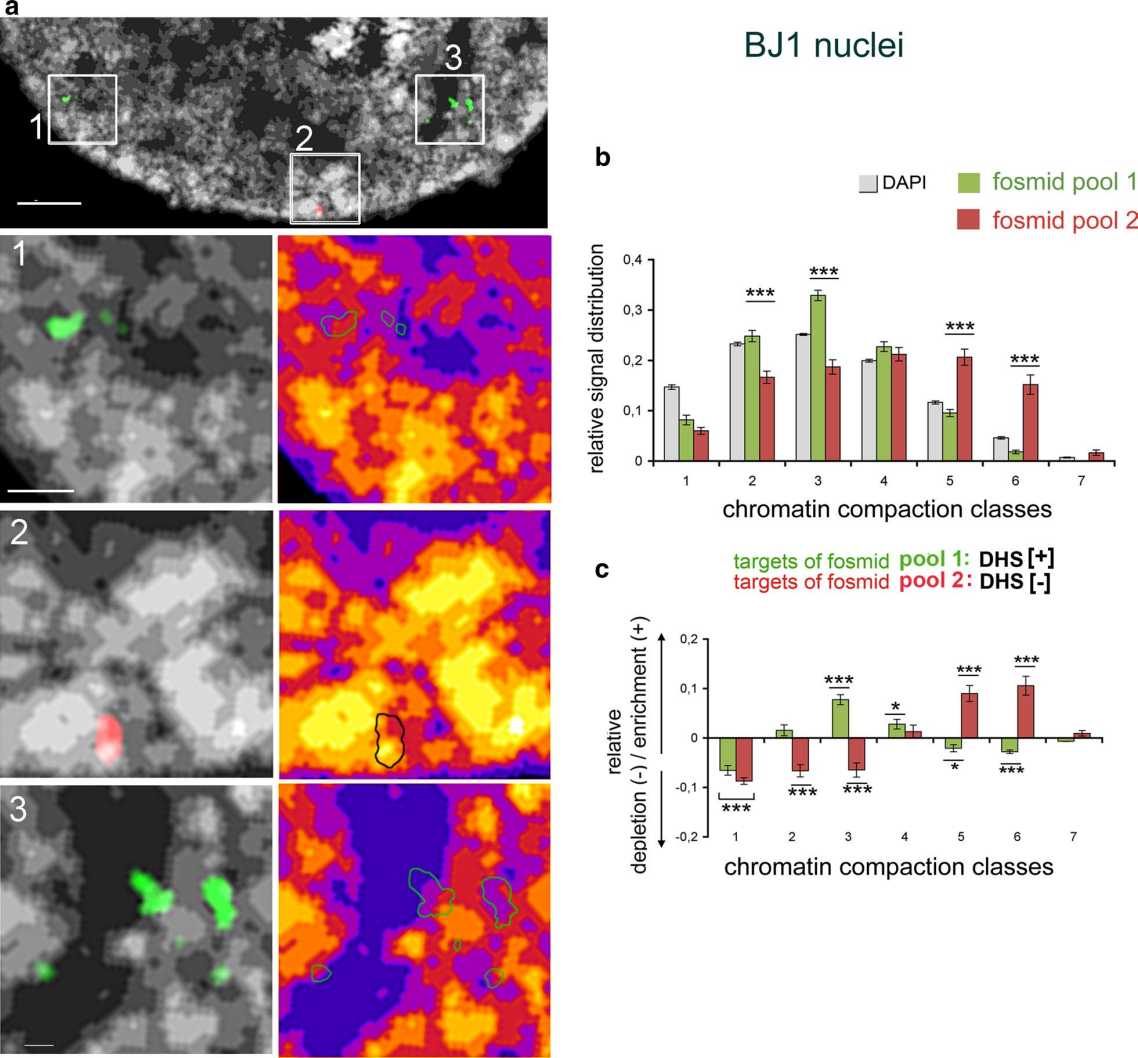
3D nuclear topography and quantitative mapping of ~40-kb targets of DHS[+] and DHS[−] regions in BJ1 nuclei. **a** Part of a SIM light-optical section from a whole nucleus acquisition with framed areas indicating representative *inset* magnifications 1–3. DAPI-stained DNA after intensity classification shown as *gray* gradations and *color* heat maps, respectively. Segmented signals delineating targets of fosmid pool 1 (DHS[+], *green*) and fosmid pool 2 (DHS[−], *red*) show a preferential localization of pool 1 signals DHS[+] in zones of low DAPI intensity and of pool 2 (DHS[−]) within the more compacted core of CDCs as shown by outlined signals in *color* heat maps: pool 1 = *green*, pool 2 = *black*). *Scale bar* 2 µm, *insets* 0.5 µm. **b** Quantified distributions (*N* = 25 nuclei) of fosmid pool 1 (DHS[+], *green*) and pool 2 (DHS[−], *red*) within respective chromatin compaction classes (all classes shown in *gray*) confirm the significantly distinct topography for DHS[+] and DHS[−] associated signals with a shift of DHS[−] sites toward higher compaction classes. **c** Quantified levels of relative enrichment (positive values) or depletion (negative values) of fosmid pool 1 and pool 2 signals within chromatin compaction classes. *Error bars* = standard deviation of the mean.*p ≤ 0.05; **p ≤ 0.01; ***p ≤ 0.001

BJ1 is a diploid cell strain (46, XY, Additional file 3). Accordingly, up to twelve distinct segments carrying DHS[+] sites can be targeted in a BJ1 nucleus by fosmid pool 1 and up to four distinct DHS[−] segments by fosmid pool 2 (Additional file 4: movie_1). Representative SIM sections of a BJ1 nucleus demonstrate the preferential localization of DHS[+] targets in low chromatin compaction classes at the periphery of CDCs, and of DHS[−] targets in more interior and compacted regions (Fig. 4a). Quantitative image analysis confirms the highly significant enrichment of DHS[+] segments indicating active TREs in low chromatin compaction classes 2 and 3 and of DHS[−] segments (inactive TREs) in high compaction classes 5 and 6 (*p* < 0.001 for classes 2/3 and 5/6) (Fig. 4b, c). This difference is consistently seen in all single-cell profiles considered as a series of ‘snapshots’ (Additional file 5).

Both fosmid pools were also hybridized on the chromosomally rearranged adenocarcinoma cell line A549. This cell line was used for comparison with BJ1 cells for two reasons: First, its DHS profile is clearly distinct from BJ1 cells in that all genomic targets delineated by fosmid pool 1 are DHS[−] in A549 (Additional file 6) indicating the lack of active TREs as supported by a lack of ‘open’ chromatin marks (Additional file 2). Second, their flat nuclear shape with *z*-diameters of 4–5 µm (data not shown) facilitates 3D-SIM acquisition [51]. Numerical and structural rearrangements in A549 cells (see Additional file 7: for karyotype) permit up to 19 distinct hybridization sites for fosmid pool 1 and up to 5 sites for fosmid pool 2 (Additional file 8: movie_2). In contrast to BJ1, A549 nuclei show a fairly similar 3D nuclear topography of targets delineated by fosmid pools 1 and 2 that are both DHS[−]. A representative A549 nucleus and quantitative evaluation of 10 nuclei (as provided in Additional file 9) show the highest enrichment of signals for both fosmid pools in chromatin compaction classes 4 and 5 (for single-cell profiles, see Additional file 10; for detailed information on statistical values for all measurements, see Additional file 11).

**Fig. 5:**
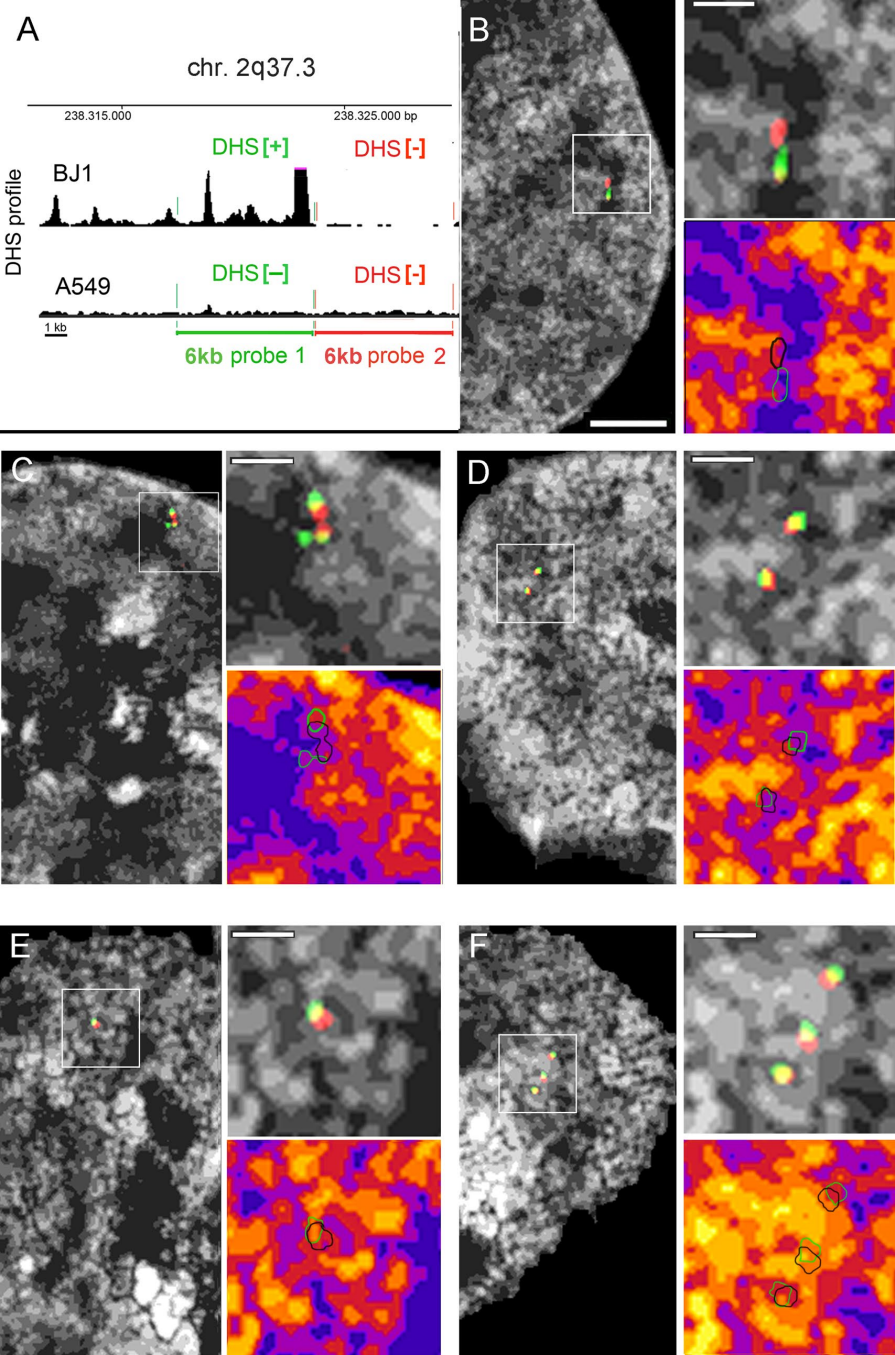
Scheme and topography of two contiguous 6-kb segments targeting a DHS[+] and adjacent DHS[−] site. **A** Scheme of probe 1 (*green*; DHS[+] in BJ1, DHS[−] in A549) and probe 2 (*red*; DHS[−] both in BJ1 and in A549 cells) in relation to their DHS profiles (*black*; *browser shots* adopted from http://encodeproject.org/). **B**–**D** SIM light-optical sections from 3D acquisitions of different BJ1 nuclei and representative *inset* magnifications delineating DAPI-stained DNA after intensity classification (shown as *gray* gradations and as *color* heat maps) and segmented probe signals (probe 1 *green*; probe 2 *red*). Signal positions are shown as outlines in the *color* heat maps (*red signals* outlined in *black* for better visibility). Magnifications reveal the localization of both *red* and *green signal*s in compartments of low chromatin compaction. Note variability of signal conformation: **B** elongated signal pair; **C** signal pair with extended *red signal* lined by two separate *green signals* suggestive for ongoing replication; **D** two compact separate signal pairs <0.5 µm apart suggest two chromatids after replication. A second signal pair (see Additional files 12, 13, 14) is seen in a different section of each nucleus. **E**, **F** Same probe setup in A549 cells shows the preferential topology of targeted sites (both DHS[−]) toward the compacted core of a chromatin domain cluster. *Scale bar* 2 µm, *inset* 0.5 µm

To dissect the topography of a specific smaller segment in BJ1 cells harboring a cluster of DHS[+] peaks over its entire length, we performed 3D-FISH with a probe delineating a ~6-kb segment on chromosome 2q37 located within the 17-kb-long first intron of the *COL6A3* gene, termed ‘6-kb probe 1’ (Fig. 5A). This segment contains several TREs such as an annotated transcription start site, enhancers and CTCF-binding sites (Additional file 2) with apparently high activity in BJ1 cells. The topography of this segment was compared in A549 cells where the respective sites are DHS[−] over the entire probe length (Fig. 5A). In all experiments, probe 1 was co-hybridized with a differently labeled probe which delineates an adjacent 6-kb segment with no known content of TREs [52] and accordingly DHS[−] status in both BJ1 and A549 cells, termed ‘6-kb probe 2’ (Fig. 5A). Co-hybridization of the two contiguous probes was performed for the following reasons: First, we wanted to test whether we could detect a distinct localization in different chromatin compaction classes of DHS[+] and DHS[−] sites at this length scale. Second, using a probe set composed of adjacent differently labeled probes for quantitative assessment helps to exclude in an unbiased way any dotted unspecific background signals, which can become a challenge for very small single-copy 3D FISH signals, reflecting with a signal volume of ~0.005 µm^3^ the volumetric resolution limit of 3D-SIM. Signals with a similar volume can arise from background fluorophores below this resolution limit. Such background cannot be entirely avoided even under most meticulous experimental conditions. Signals were accepted as true hybridization events only in case of centroid distances ≤500 nm between green and red signals, taking into account that the length of 10 kb as a fully extended 10-nm fiber is approximately 500 nm [53].

**Fig. 6:**
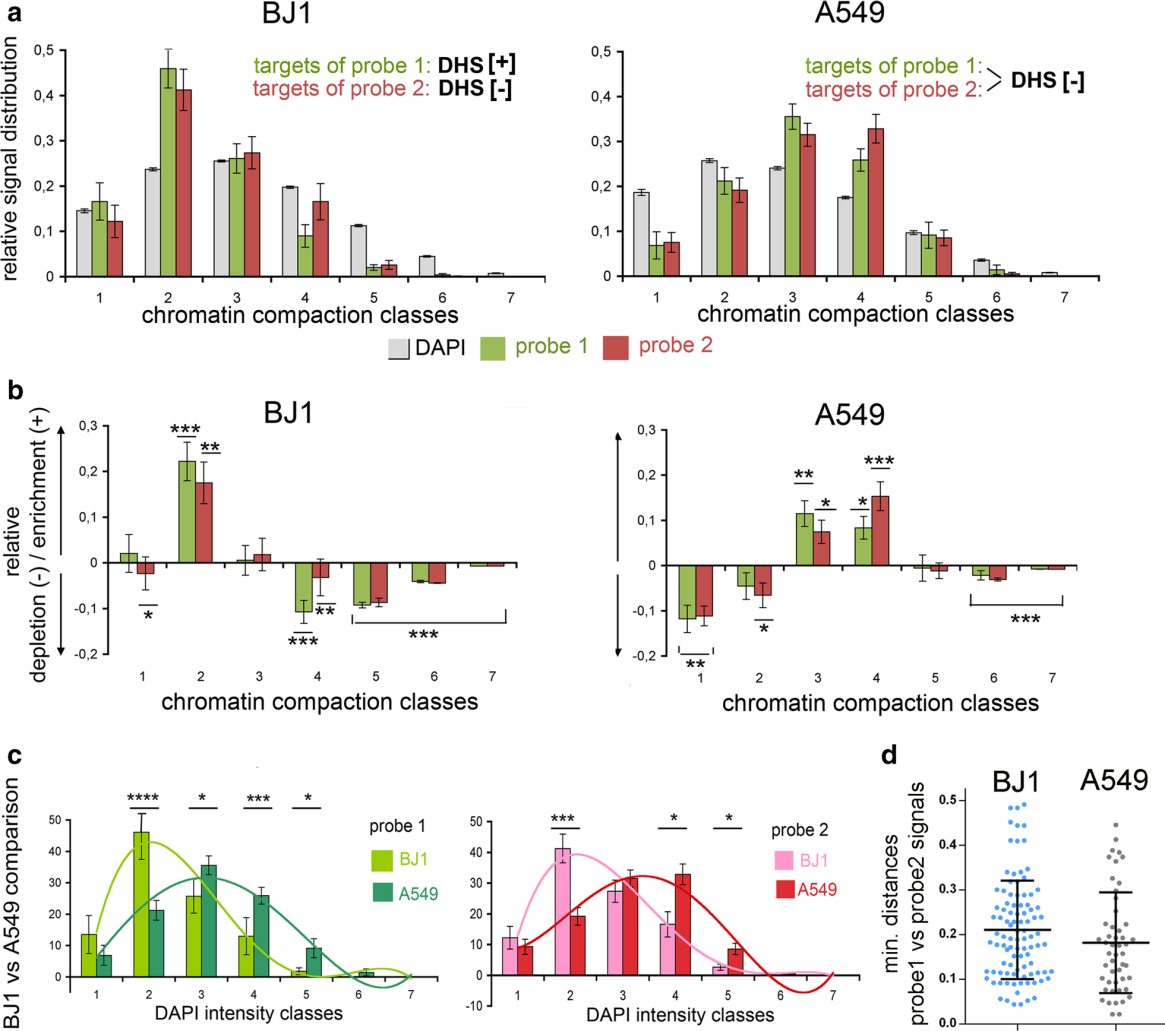
Quantitative 3D mapping of two contiguous 6-kb DHS[+] and DHS[−] targets on DAPI intensity classes. **a** Relative signal distribution of probe 1 (*green*) and probe 2 (*red*) on respective DAPI intensity classes (all classes shown in *gray*) in BJ1 (*left; N* = 20) and A549 nuclei (*right*, *N* = 10). Note similar distribution patterns for both probes (n.s. at *p* < 0.5) within a cell type but distinct distribution between cell types. **b** Quantified levels of relative enrichment/overrepresentation (positive values) or depletion/underrepresentation (negative values) of probe signals relative to the classified DAPI signals show a highly significant overrepresentation in low-density class 2 and an underrepresentation in high-density classes 4–6. In A549 cells, both probes are significantly underrepresented in low-density classes 1 and 2 and overrepresented in classes 3–4. *Error bars* standard deviation of the mean. **c** Comparison between BJ1 (*light green/light red*) and A549 (*dark green/dark red*) confirms the distinct topography of both probes in distinct chromatin density compartments. *Error bars* standard deviation of the mean. **d** Minimal distances (nearest-neighbor analysis) between all differently labeled fluorescent signals for probe 1 and probe 2 in BJ1 and A549 cells, n.s. at *p* < 0.5 level. *p ≤ 0.05; **p ≤ 0.01; ***p ≤ 0.001

Representative SIM sections of hybridized BJ1 (Fig. 5B–D) and A549 nuclei (Fig. 5E, F) reveal signals with a lateral diameter of ~120–150 nm. This length is at the diffraction limit of 3D-SIM, so their true diameters may be smaller. In BJ1 nuclei, both signal pairs of 6-kb probes 1 and 2 are consistently noted in low chromatin compaction zones at the periphery of chromatin domain clusters (CDCs). In A549 nuclei where both probes delineate DHS[−] sites, signals are closer toward the compact core of CDCs, yet excluded from the most compact zones in the interior of CDCs. Examples shown in Fig. 5C, D are suggestive of replicated DNA in close proximity (C) or separated by ~400 nm (D), since a second site of (replicated) signal pairs is seen elsewhere in these nuclei (Additional files 12, 13, 14: movies_3, 5; for a presumable G1 nucleus, see Additional file 15: movie_6). Replicated signal pairs hint to a consistent orientation since 3D distances between centroids of 6-kb probe 1 signals are found significantly smaller compared to 6-kb probe 2 centroid distances in both cell types (Additional file 16). Quantitation in BJ1 cells confirms enrichment of the 6-kb DHS[+] segment delineated by probe 1 in the low chromatin compaction class 2 and a depletion in classes 4–6 (Fig. 6a, b left panels), while the corresponding (DHS[−]) segment in A549 cells shows a significant enrichment in higher compaction classes (Fig. 6a, b right panels, Fig. 6c shows the direct comparison between BJ1 and A549 cells). Notably—except for few nuclei (see Additional file 17: for single-cell profiles)—probe 2 largely mirrors the 3D topography of probe 1 in both cell types. This was expected for A549 cells where both probes delineate DHS[−] segments. In BJ1 cells, the similar topography of both segments within the ANC may be explained as a passive consequence of the functionally important looping out of a DHS[+] segment with an active TRE enforcing the concomitant movement of an adjacent DHS[−] segment at this length scale. Minimal distance measurements between centroids of 6-kb probe 1 and 2 signals show a slightly higher, although not significant extension of the entire ~12-kb segment in BJ1 compared to A549 nuclei (Fig. 6d). This finding may suggest a difference in chromatin compaction of the respective target sites in the two cell lines. A detailed information on statistical values for all measurements is given in Additional file 18.

## Discussion

In this study, we further explored the structural and functional organization of chromatin domain clusters (CDCs) at the level of super-resolution microscopy and compared for the first time the 3D nuclear topography of selected DHS[+] and DHS[−] sites which typically reflect active or inactive TREs, respectively. In two human types of cultured cells (BJ1 and A549), we found an enrichment of active TREs (DHS[+]) in the ANC, i.e., at the periphery of CDCs extending into the IC, whereas inactive TREs (DHS[−]) were enriched toward the more compacted interior of CDCs, constituting the ‘inactive’ nuclear compartment (INC). Further studies with other cell types and species are indicated to test whether our current results present a general feature of the ANC–INC model, which was based on studies of a variety of normal and cancer cell types from different mammalian species [43, 47, 48]. In case that the ANC–INC model stands further experimental scrutiny, the spatial separation of inactive TREs in the INC could be explained as additional protection against their inappropriate activation, but experimental evidence for this is lacking. The distinctly different topography of active and inactive TREs in the ANC and INC, respectively, suggests a dynamic organization of CDCs, which allows positional changes of TREs between the two compartments depending on their functional state.

### Structural and functional organization of CDCs

Our study indicates a radially arranged structural organization of CDCs based on layers of different chromatin compaction with most decondensed, transcriptionally competent chromatin at the CDC periphery to the most compact chromatin within the CDC interior. This layered organization suggests transition zones of chromatin compaction between CDs with a fully ‘closed’ and a fully ‘open’ configuration [6, 54, 55]. Hi-C experiments revealed two higher-order compartments A and B, respectively, of ~1-Mb CDs corresponding to open (transcriptionally competent) and closed (transcriptionally silent) chromatin [33], but their relationship to CDCs has remained elusive. Current microscopic evidence demonstrates a significant enrichment of transcriptionally competent chromatin at the periphery and of repressed chromatin in interior of CDCs [43, 47, 48]. The term INC suggests a heterochromatic nature of compact CDs. According to the classical view of heterochromatin, facultative heterochromatic contains repressed genes, while constitutive heterochromatin is built up from repetitive blocks without interspersed coding genes. In both cases, the paucity of transcription was formerly considered as a hallmark of heterochromatin. Recent evidence, however, has shown that non-coding RNAs, called hetRNA, are transcribed from heterochromatin at a previously unexpected level, including pericentric and intergenic major satellite repeats [56].

Our initial assessment of the extent of minimal movements required for shifts of regulatory sequences between the decondensed periphery of CDCs and the compact interior shows that positional changes <100 nm may suffice. However, covered distances may be substantially larger since movements of genomic elements within the nuclear landscape typically occur along individual trajectories resembling an anomalous diffusion rather than the shortest possible path [19]. A considerable local dynamic of nucleosomes in living cells that drive chromatin accessibility was reported [57]. An in-depth exploration of the dynamic nature of CDCs, including movements of CDs and chromatin loops, requires live cell imaging with resolution <100 nm. The necessity to study large numbers of sites of specific types of TREs with special scrutiny of housekeeping, developmental and cell-type-specific genes adds another methodological challenge for future studies.

### Perspectives of a functional interplay between CDCs and IC channels

3D/4D super-resolution microscopic studies are limited by the fact that only a few targets can be studied in each experiment. For our initial study, we selected eight targets on seven chromosomes (compare Fig. 3) and altogether mapped the 3D positions of about 300 DHS[+] and 400 DHS[−] sites within a total of 45 BJ1 and 30 A549 cell nuclei. Hi-C of cell populations with millions of fixed cells allows the genome-wide detection of billions (10^9^) of pairwise 3D DNA–DNA contacts yielding a 1-kb resolution of topologically associating domains (TADs) [32, 33, 36]. In line with microscopic evidence, Hi-C led to the discovery of ~1-Mb-sized TADs carrying smaller sub-TADs and larger meta-TADs [32, 36, 37]. In contrast to microscopic studies, Hi-C provides the major advantage of direct comparisons with other genome-wide data sets, such as gene expression profiles, histone signatures or DHS sites (see, e.g., Roadmap Epigenomics Mapping Consortium data). Recent advancements of Hi-C have made it possible to explore the 3D organization of the entire genome in individual cell nuclei as well, although at the cost of a strongly reduced number of 3D contact sites detected in individual cell nuclei compared with Hi-C of cell populations [24, 58].

Considering these powerful possibilities, one may argue that Hi-C may replace microscopic studies, which is, however, not the case as outlined below. Hi-C experiments support a CT organization based on a 3D multiloop aggregate/rosette chromatin architecture [59]. At face value such an organization may leave sufficient inter-chromatin space to allow diffusion of macromolecules directly through open loops. Maeshima and colleagues have challenged this view with their proposal of a liquid drop model of chromatin domain organization [60, 61]. According to this model, chromatin domains are composed of irregularly folded, highly compacted 10-nm nucleosome fibers. The diffusion of non-coding RNAs and of single transcription factors into the interior of these compact CDs is possible, yet highly constrained [62]. Based on Monte Carlo simulations, the authors have proposed that small gene-specific transcription factors with a size of ~50 kD can penetrate into compact chromatin domains and search their target sequences, whereas large transcription complexes are excluded.

Taking into account a high level of compaction, the ANC–INC model [21] predicts that transcription factors and other functional proteins, which enter the nucleus via nuclear pores, reach their sites of action preferentially by constrained diffusion along routes provided by IC channels, which start at nuclear pores and pervade the nuclear interior between CDCs with finest branches extending into their interior. Similarly, the IC-channel network may serve for the rapid intranuclear distribution of mobile, non-coding RNAs involved in gene expression and for the export of mRNAs. Our observation of a shell-like organization of CDCs argues for the possibility that CDs with different chromatin compaction levels coexist in individual CDCs. Further studies are necessary to explore the diffusional constraints inflicted by the true physical compaction of individual CDs and TADs, respectively, as well as by structural entities, which may provide ‘stumbling blocks’ within open loops [21]. We expect that electron and super-resolved fluorescence microscopy will remain the methods of choice to analyze the true 3D geometry of CDCs, including the size, shape and extent of compaction or decondensation of individual CDs. Ongoing efforts to achieve multicolor visualization of specific DNA targets in live cells will help to address dynamic aspects of CDC organization [28, 63].

## Conclusions

This study demonstrates the non-random distribution of active and inactive transcription regulatory elements (TREs) within the higher-order chromatin landscape of cell nuclei studied in a diploid human fibroblast line (BJ1) and an aneuploid human lung cancer cell line (A549). Data were obtained by 3D FISH with differently labeled DNA probe sets targeting sites with apparently active or inactive TREs and 3D quantitative image analyses of DAPI-stained nuclei recorded with 3D structured illumination microscopy (3D-SIM). Our results indicate a 3D organization of chromatin domain clusters (CDCs) with radially arranged layers of increasing chromatin compaction from the periphery toward the CDC core. Segments harboring active TREs are significantly enriched at the decondensed periphery of CDCs, while segments with inactive TREs are embedded within the more compacted interior layers. This difference suggests positional changes of TREs within CDCs depending on their functional state. Live cell studies with high resolution are required to directly observe a relocation of TREs within CDCs in line with their state of activity. A further improvement of resolution beyond the reach of SIM, achieved, for example, by single-molecule localization microscopy [64] is essential both with respect to the precision of target localization and with respect to the quantitative measurements of 3D chromatin domain compaction within CDCs, which depends critically on the resolution limit. Comparisons of individual cells in their living state and after 3D-FISH are further necessary to quantify at a given resolution the extent of potential changes of the 3D chromatin landscape due to fixation and DNA denaturation required for 3D-FISH [30, 65].

## Materials and methods

### Cells and culture conditions

BJ1-tert skin fibroblasts (ATCC # CRL-2522) and A549 cells Human Lung Carcinoma Epithelial Cells (ATCC #: CCL-185) were grown under conditions used for DNase-seq analysis. BJ1 cells: MEM, supplemented with 1.5 g/L sodium bicarbonate, 1 mM sodium pyruvate, 2 mM l-glutamine, 1× non-essential amino acids, 10% FBS, Pen-Strep (1×). A549 cells: F-12 K Medium, supplemented with 10% FBS, Pen-Strep (1×).

### Generation of complex DNA probe sets

DHS profiles in BJ1 and A549 cells were identified on availability of NIH Roadmap Epigenomics Mapping Consortium data (www.roadmapepigenomics.org hg19). Genomic segments with or void of DHS[+] sites in BJ1 and A549 cells, respectively, were selected as DNA targets for the generation of two probe sets. Fosmid clones for fosmid pools 1 and 2 are described in Fig. 3. For optimal target representation, a pair of overlapping fosmid clones was selected for each region from the WIBR-2 human fosmid library (http://www.ncbi.nlm.nih.gov/clone/library/genomic/) and purchased from the BACPAC Resources Center. For clone ID and genomic positions, see Additional file 19. In total, 6-kb probes 1 and 2, located within fosmid clone G248P85778F6 (as detailed description in Fig. 5A), were generated by PCR of 1-kb subfragments and subsequent pooling of amplification products (for position and primer_seq, see Additional file 20). PCR was performed in 10 mM Tris–HCl with pH 8.3, 50 mM KCl, 2 mM MgCl_2_, 200 µM each dNTP, 1 µM each primer, 10 ng template DNA using 25 cycles of 94 °C for 30 s/56 °C for 30 s/72 °C for 1 min. Equal amounts of fosmid DNA- or PCR-amplified DNA assigned for labeling with either biotin or digoxigenin in the respective probe set were pooled and the pooled samples labeled by either biotin or digoxigenin by standard nick translation. Forty nanograms of labeled probe together with 20-fold excess of human COT-1 DNA was dissolved per 1 µl hybridization solution (2 × SSC/10% dextran sulfate/50% formamide). Hybridization efficiency and specificity of probes were verified on human metaphase chromosomes (Additional file 21).

### Regulatory element annotation

The following data sets were converted from hg19 into hg38 using the liftover chain downloaded from the UCSC web-page and the liftover-tool implemented in the R package rtracklayer.

To identify regulatory elements, we accessed the Ensembl Regulation Database (v 88) [52] via the R package biomaRt. We used the data sets of Human Other Regulatory Regions, which only identified FANTOM annotations [49] as well as Human Regulatory Features to find annotated regulatory elements and Human Regulatory Evidence to get a better idea, what kind of chromatin marks or binding sites were found [66]. Type and sequence location of TREs targeted by targeted in this study are summarized in Additional file 2.

### Pretreatment of cells for 3D-FISH and hybridization/detection setup

Unsynchronized cells grown up to ~60–70% confluency on high-precision borosilicate glass coverslips (170 ± 5 µm thickness) were fixed in 4% *para*-formaldehyde/PBS (10 min) followed by a stepwise replacement with PBS/0.05% Tween 20 and subsequent quenching of free aldehydes by 20 mM glycine (10 min). Cell and nuclear membranes were permeabilized by 0.5% Triton X-100/PBS (10 min), repeated freezing/thawing of cells in liquid N2 and subsequent gradual incubation in 0.1 N HCl (5 min). Cells were equilibrated in 2× SSC; at this step, RNA was removed by RNAse I treatment (100 μg/ml, 1 h at 37°). Cells were incubated in 50% formamide/2× SSC (pH = 7.0) at 4 °C until hybridization, at least overnight.

After simultaneous denaturation of cells and probes (76°/2 min), hybridization was performed at 37 °C for at least 48 h. Stringent washings in 0.1× SSC at 60 °C were followed by extensive blocking in 2% bovine serum albumin/0.5% Fish gelatin/4× SSCT for at least 2 h at RT. Probe detection was performed with avidin-Alexa488 (Molecular probes) and mouse-antidigoxigenin (Sigma) followed by an Alexa594-conjugated anti-mouse IgG (Molecular Probes). Cells were postfixed for 10 min in 4% formaldehyde/PBS, and DNA was counterstained with 1 µg/ml 4′,6-diamidino-2-phenylindole (DAPI) in 2× SSC. Samples were mounted in Vectashield antifade mounting medium (Vector Lab). A detailed protocol for 3D-FISH meeting the requirements for 3D-SIM is provided in [42].

### 3D-SIM

Super-resolution structured illumination imaging was performed on a DeltaVision OMX V3 system (Applied Precision Imaging/GE Healthcare) equipped with a 100×/1.4 UPlan S Apo oil immersion objective (Olympus), Cascade II:512 EMCCD cameras (Photometrics) and 405, 488 and 593 nm lasers (for detailed description, see [67]). Raw data image stacks were acquired with 15 raw images per plane (5 phases, 3 angles) and an axial distance of 125 nm and then computationally reconstructed with a Wiener filter setting of 0.002 and channel-specific optical transfer functions (OTFs) using SoftWoRx (Applied Precision). The reconstruction process generates 32-bit data sets with the pixel number doubled in the lateral axes, resulting in the pixel size being halved from 79–39.5 nm in order to meet the Nyquist sampling criterion. The level of spherical aberration was minimized and matched to the respective OTFs using immersion oil of different refractive indices (RI). Best results were typically obtained with OTFs measured on red, green (both 110 nm diameter) and blue (170 nm diameter) fluorescent FluoSpheres (Invitrogen) using RI 1.512, and sample acquisition with RI 1.512 for depth adjustment in the region of optimal reconstruction a few µm into the sample. Images from the different color channels were corrected for chromatic aberration in SoftWoRx with alignment parameters obtained from calibration measurements with 0.2-µm-diameter TetraSpeck beads (Invitrogen). To normalize all image stacks for subsequent image processing and data analysis, the original 32-bit images were shifted to positive values and transformed to 16-bit. All further image processing was performed in ImageJ (http://rsb.info.nih.gov/ij/). For a detailed description of methodological image quality assessment survey, see [68].

### Chromatin compaction classification by 3D assessment of DAPI intensity classes

Nuclei voxels were identified automatically from the DAPI channel intensities using Gaussian filtering and automatic threshold determination. For chromatin quantification, a 3D mask was generated in ImageJ to define the nuclear space considered for the segmentation of DAPI signals into seven classes with equal intensity variance by an in-house algorithm described previously [43, 48], available on request. Briefly, a hidden Markov random field model classification was used, combining a finite Gaussian mixture model with a spatial model (Potts model), implemented in the statistics software R [69, 70]. This approach allows threshold-independent signal intensity classification at the voxel level, based on the intensity of an individual voxel. Color or gray value heat maps of the seven intensity classes in individual nuclei were performed in ImageJ. For a detailed description, see [46].

### Semi-automatic segmentation of hybridization signals and their allocation on 3D chromatin compaction classes

Individual voxels of FISH signals of the respective marker channels were segmented using a semi-automatic thresholding algorithm (using custom-built scripts for the open-source statistical software R http://www.r-project.org, available on request). Xyz-coordinates of segmented voxels were mapped to the seven DNA intensity classes. The relative frequency of intensity weighted signals mapped on each DAPI intensity class was used to calculate the relative distribution of signals over chromatin classes. For 3D mapping of two contiguous differentially labeled 6-kb DNA probes (6-kb probes 1 and 2) relative to chromatin compaction classes, any fluorescent dot with a distance >0.5 µm from the nearest signal centroid of a differentially labeled target was attributed to background and eliminated from further consideration after signal segmentation with appropriate parameter settings. For each studied nucleus, the total number of voxels counted for each intensity class and the total number of voxels identified for the respective FISH signals were set to 1.

As an estimate of over/under representations (relative depletion/enrichment) of marker signals in the respective intensity classes, we calculated the difference between the percentage points obtained for the fraction of voxels for a given DAPI intensity class and the corresponding fraction of voxels calculated for the FISH signals. Calculations were performed on single-cell level and average values over all nuclei used for evaluation and plotting. For a detailed description, see [46].

### Nearest-neighbor/minimal distance measurements

Nearest-neighbor/minimal distance measurements between centroid xyz coordinates of differently labeled segmented FISH ‘objects’ were taken using the TANGO Plugin for ImageJ/Fiji [43, 71, 72]. Mode-subtracted, 16-bit transformed 3D-SIM image stacks were imported into TANGO. Xyz centroid coordinates from segmented objects were extracted based on the geometrical gravity center of the segmented 3D foci and subsequently used for centroid mapping and nearest-neighbor analysis. Nearest-neighbor distances of different experiments were analyzed by pairwise *t* test comparison with Bonferroni correction of level of significance. For the measurements of minimal absolute distances between DAPI intensity classes, distances between voxels were calculated from their centroid. For each class, the distance from a voxel of this class to the nearest voxel of each other class was calculated.

### Statistical evaluation

GraphPad Prism 6 was used for plots and statistical evaluations. Statistical differences were tested using the Wilcoxon rank sum test with continuity correlation as well as Student’s *t* test (two-tailed, *p* < 0.05). For interpolation models of DHS[+] and DHS[−] distributions, a second-order polynomial fit was used. SD and SEM were used for error bars, as indicated.

### Data access

DHS profiling: www.roadmapepigenomics.org hg19

Fosmid library: http://www.ncbi.nlm.nih.gov/clone/library/genomic/

## Additional files

**Additional file 1.** ANC–INC network model of nuclear organization based on spatially co-aligned active and inactive nuclear compartments (for detailed information, see [21]). Nuclear organization according to co-aligned 3D networks of an active (ANC) and an inactive nuclear compartment (INC). The ANC is a composite structural and functional entity of a 3D-channel network, the ‘Inter-chromatin-compartment’ (IC) together with the decondensed periphery of a higher-order chromatin network, which is built up from ~1-Mb chromatin domains (CDs), representing basic units of larger chromatin domain clusters (CDCs). The decondensed periphery of CDCs is known as the perichromatin region (PR). According to this model, the PR harbors regulatory and coding sequences of active genes and represents the preferential nuclear subcompartment for transcription, RNA-splicing, and possibly also for DNA replication and repair. Small chromatin loops expand from the perichromatin region into the interior of IC channels which start/end at nuclear pore complexes. Nuclear bodies are located within the IC, which serves as a transport system for macromolecule complexes. The INC is represented by the compacted core of CDCs enriched in markers for silent chromatin (Fig. modified from [21]).

**Additional file 2.** Overview of type, sequence location and open chromatin marks of TREs targeted by probe sets used in this study (fosmid pools 1 and 2; 6-kb probes 1 and 2). Data are based on [52]. For the segment covered by 6-kb probe 2, no TREs were identified in the used databases. Sequence coordinates represent the coordinates for hg38 after conversion of hg19 into hg38 by liftover. The used databases do not provide data on ‘open chromatin marks’ H3K9ac and H3K4me3 as additional indirect information for their state of activity at the respective loci in BJ1 cells. In the sheet ‘open chromatin marks,’ available data for IMR90 fibroblasts are shown instead. IMR90 cells show an almost identical DHS[+] profile to BJ1 cells (www.roadmapepigenomics.org); accordingly, similar epigenetic signatures between both cell lines can be assumed.

**Additional file 3.** Metaphase spreads of BJ1 cells: Five out of six randomly selected Giemsa stained metaphase spreads reveal an inconspicuous diploid chromosome set of n = 46, XY (n = 45 in metaphase 2 is likely due to loss of one chromosome during preparation).

**Additional file 4.** Movie_1 entire image stack of a BJ1 nucleus hybridized with fosmid pools 1 and 2. DAPI-stained DNA after intensity classification shown in gray. Fosmid pool 1 (green), fosmid pool 2 (red).

**Additional file 5.** Single-cell profiles for target sites of fosmid pools 1 and 2 mapped to chromatin compaction classes in BJ1 cells for illustration of intercellular variability. (**A**) Mapping profiles from ten randomly chosen individual nuclei illustrate consistent distinct distribution profiles of fosmid pool 1 (green) toward low chromatin compaction classes and of pool 2 (red) toward higher chromatin compaction classes. **(B)** Standard deviations of relative probe signal distributions of all evaluated nuclei (compare Fig. 4 for standard errors of the mean (SEM). **(C)** Standard deviations of DAPI signal distribution on classes (compare Fig. 4 for SEM).

**Additional file 6.** DHS profiles and fosmid clones used for target regions delineating DHS[−] sites on different chromosomes in A549 nuclei. **(A)** Selected regions with clones of fosmid pool 1 (green) and **(B)** with clones of fosmid pool 2 (red). DHS profile in black (browser shots adopted from http://encodeproject.org/). Note: probe sets are identical to probe sets shown in Fig. 3.

**Additional file 7.** M-FISH karyotype analysis of A549 cells. **(A)** Representative karyotype obtained by M-FISH after combinatorial labeling of chromosome-specific paint probes with seven fluorochromes. **(B)** Quantitation of 20 metaphases reveals a karyotype with 62–66 chromosomes with consistent structural rearrangements involving chromosomes 1,2,3,6,8,11,15,19,20. This constellation allows for fosmid pool 1 up to 19, for fosmid pool 2 up to five distinct hybridization sites in a nucleus (compare Additional file 6).

**Additional file 8.** Entire image stack of A549 nucleus after hybridization with fosmid pools 1 and 2. DAPI-stained DNA after intensity classification shown in gray. Fosmid pool 1 (*green*), fosmid pool 2 (*red*).

**Additional file 9.** 3D nuclear topography and quantitative mapping of ~40 kb targets of DHS[−] regions in A549 nuclei. **(A)** Part of a SIM light-optical section from a whole nucleus acquisition with representative inset magnifications. DAPI-stained DNA after intensity classification shown as gray gradations and color heat map, respectively. Segmented signals delineating targets both of fosmid pool 1 (*green*) and fosmid pool 2 (*red*) show a similar location with regard to chromatin compaction classes (asterisks in color heat maps, pool 1 (*green*), pool 2 (*black*). Scale bar 2 µm, insets 0.5 µm. **(B)** Quantified distributions (N = 10 nuclei) of fosmid pools 1 (*green*) and 2 (*red*) within respective chromatin compaction classes (all classes shown in gray). **(C)** Quantified levels of relative enrichment (positive values) or depletion (negative values) of fosmid pool 1 and pool 2 signals show an enrichment of signals in higher compaction classes. Error bars = standard deviation of the mean *p ≤ 0.05, **p ≤ 0.01, ***p ≤ 0.001.

**Additional file 10.** Single-cell profiles for target sites of fosmid pools 1 and 2 mapped to chromatin compaction classes in A549 cells for illustration of intercellular variability. **(A)** Mapping profiles of A549 nuclei (N = 10) illustrate for most nuclei fairly similar distribution profiles of fosmid pool 1 (*green*) and fosmid pool 2 (*red*). **(B)** Standard deviations of relative probe signal distributions of all evaluated nuclei (compare Additional file 8 for standard errors of the mean (SEM). **(C)** Standard deviations of DAPI signal distribution on classes (compare Additional file 8 for SEM).

**Additional file 11.** Significance values for relative signal distributions of fosmid pools 1 and 2 in BJ1 and A549 cells.

**Additional file 12.** Entire image stack of the nucleus shown in Fig. 5B. DAPI-stained DNA after intensity classification (blue). Segmented probe signals (green/red, seen in section z19 and 23) reveal two sites of double signal pairs, suggestive of ongoing/post-replication. Note that the respective section shown in Fig. 5B is horizontally flipped for arrangement.

**Additional file 13.** Entire image stack of the nucleus shown in Fig. 5C. DAPI-stained DNA after intensity classification (blue). Segmented probe signals (green/red, seen in sections z11 and 15) reveal two sites of double signal pairs, suggestive of ongoing/post-replication.

**Additional file 14.** Entire image stack of the nucleus shown in Fig. 5D. DAPI-stained DNA after intensity classification (blue). Segmented probe signals (green/red seen in sections z27 and 34) reveal two sites of double signal pairs, suggestive of ongoing/post-replication.

**Additional file 15.** Entire image stack of a presumable G1 nucleus. DAPI-stained DNA after intensity classification (blue). Segmented probe signals (green/red seen in sections z24 and 35) reveal two sites of a single signal pair, suggestive of G1.

**Additional file 16.** 3D distances between centroids of 6-kb probe 1 (green) and centroids of 6-kb probe 2 in BJ1 (left) and in A549 cells (right). Distance measurements are restricted to distances <500 nm presumably comprising only sister chromatids of S/G2 nuclei. The smaller distances between centroids of green signals (probe 1; DHS[+] in BJ1 cells, DHS[−] in A549 cells) compared to distances between centroids of red signals (probe 2; DHS[−] both in BJ1 and A549 cells) hint to a consistent orientation of these segments irrespective of DNAse I sensitivity.

**Additional file 17.** Single-cell profiles of 6-kb probes 1 and 2 targets mapped to chromatin compaction classes in BJ1 (**A**–**C**) and A549 cells (**D**–**F**) for illustration of intercellular variability. **(A)** Mapping profiles from ten randomly chosen individual BJ1 nuclei for illustration of intercellular variabilities and similarities of relative signal distribution of probe 1 (green) and probe 2 (red) within DAPI intensity classes. Note an only marginal signal representation in classes 5–7. **(B)** Standard deviations of relative probe signal distributions of all evaluated nuclei (compare Fig. 6 for standard errors of the mean (SEM). **(C)** Standard deviations of DAPI signal distribution on classes (compare Fig. 6 for standard errors of the mean (SEM). **(D)** Respective mapping profiles from 10 individual A549 nuclei of relative signal distribution of probe 1 (green) and probe 2 (red) within DAPI intensity classes. Profiles show an overall broader distribution range compared to BJ1 nuclei. **(E)** Standard deviations of relative probe signal distributions of all evaluated nuclei (compare Fig. 6 for standard errors of the mean (SEM). **(F)** Standard deviations of DAPI signal distribution on classes (compare Fig. 6 for standard errors of the mean (SEM).

**Additional file 18.** Significance values for relative signal distributions of 6-kb probes 1 and 2 in BJ1 and A549 nuclei.

**Additional file 19.** fosmid ID (G248 library #) and sequence alignment of fosmids used. Data are based on hg19.

**Additional file 20.** Position and primer sequences used for amplification of ~1-kb subfragments for assembling of 6-kb probes 1 and 2. Data are based on hg19.

**Additional file 21.** FISH of fosmid pairs on normal human metaphases for verification of specificity. (**A)** Human metaphase ideogram with marked positions of tested fosmids. **(B**–**E)** Metaphase spreads after FISH with **(B)** fosmid pairs G248P8092D1/G248P89035F6 mapped on 1p and G248P80020B1/G248P8977D10 mapped on 12q, **(C)** G248P83004C6/G248P82547F4 mapped on 2p and G248P87313E8/G248P85778F6 mapped on 2q, **(D)** G248P8631F6/G248P88483C3 mapped on 3p and G248P87150D8/G248P89650D7 mapped on 5q, **(E)** G248P83624H8/G248P83627E4 mapped on 3p and G248P80223H2/G248P84663H7 mapped on 13q. All tested probes show a specific hybridization signal at the expected chromosomal position.

## Abbreviations

3D FISH: 3D fluorescence in situ hybridization
3D SIM: 3D structured illumination microscopy
ANC/INC: active/inactive nuclear compartment
CT: chromosome territory
CD(C): chromatin domain (cluster)
CTCF: CCCTC-binding factor
DAPI: 4′,6-diamidino-2-phenylindole
DHS[+]: DNAse I hypersensitive site indicates the presence of active regulatory sequences
DHS[−]: DNAse I non**-**sensitive site in a given cell, although the site is DHS[+] in at least one other cell type or cell line, indicates the presence of inactive regulatory sequences (exception: probe 2 delineating a DHS[−] segment lacks the content of TREs)
DNase-seq: DNAse I digestion and sequencing
Hi-C: chromosome conformation capturing combined with deep sequencing
IC: interchromatin compartment
OTF: optical transfer function
PR: perichromatin region
TAD: topologically associating domain
TF: transcription factor
TRE: transcription regulatory element
