# Supplementary figures and images for: Initial high-resolution microscopic mapping of active and inactive regulatory sequences proves non-random 3D arrangements in chromatin domain clusters
Source: Epigenetics Chromatin. 2017 Aug 7;10:39. doi: 10.1186/s13072-017-0146-0 (PMC5547466; doi:10.1186/s13072-017-0146-0)

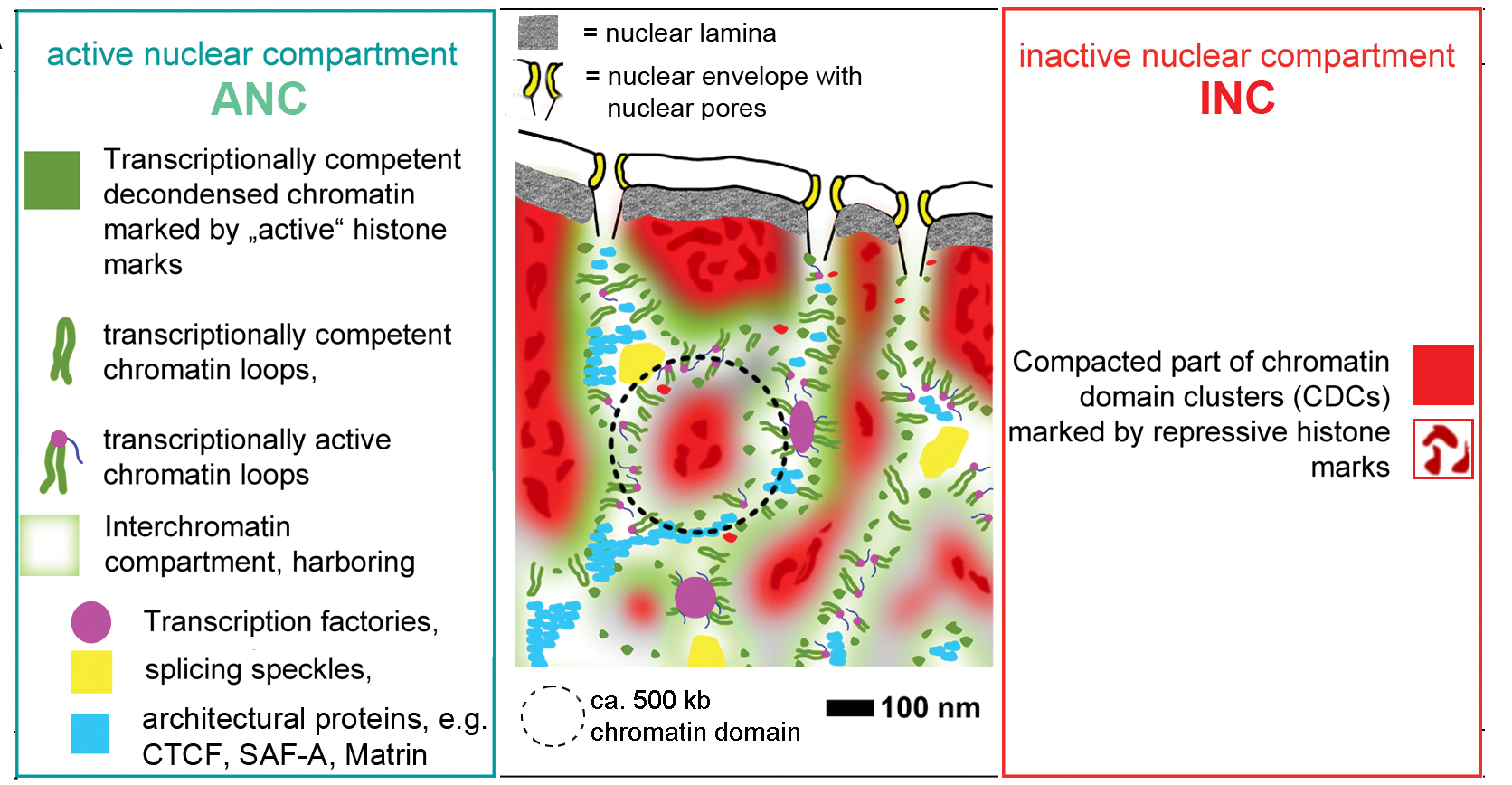

Supplement: Supplementary file 1 — Additional file 1. ANC–INC network model of nuclear organization based on spatially co-aligned active and inactive nuclear compartments (for detailed information, see [21]). Nuclear organization according to co-aligned 3D networks of an active (ANC) and an inactive nuclear compartment (INC). The ANC is a composite structural and functional entity of a 3D-channel network, the ‘Inter-chromatin-compartment’ (IC) together with the decondensed periphery of a higher-order chromatin network, which is built up from ~1-Mb chromatin domains (CDs), representing basic units of larger chromatin domain clusters (CDCs). The decondensed periphery of CDCs is known as the perichromatin region (PR). According to this model, the PR harbors regulatory and coding sequences of active genes and represents the preferential nuclear subcompartment for transcription, RNA-splicing, and possibly also for DNA replication and repair. Small chromatin loops expand from the perichromatin region into the interior of IC channels which start/end at nuclear pore complexes. Nuclear bodies are located within the IC, which serves as a transport system for macromolecule complexes. The INC is represented by the compacted core of CDCs enriched in markers for silent chromatin (Fig. modified from [21]). [file 13072_2017_146_MOESM1_ESM.jpg]

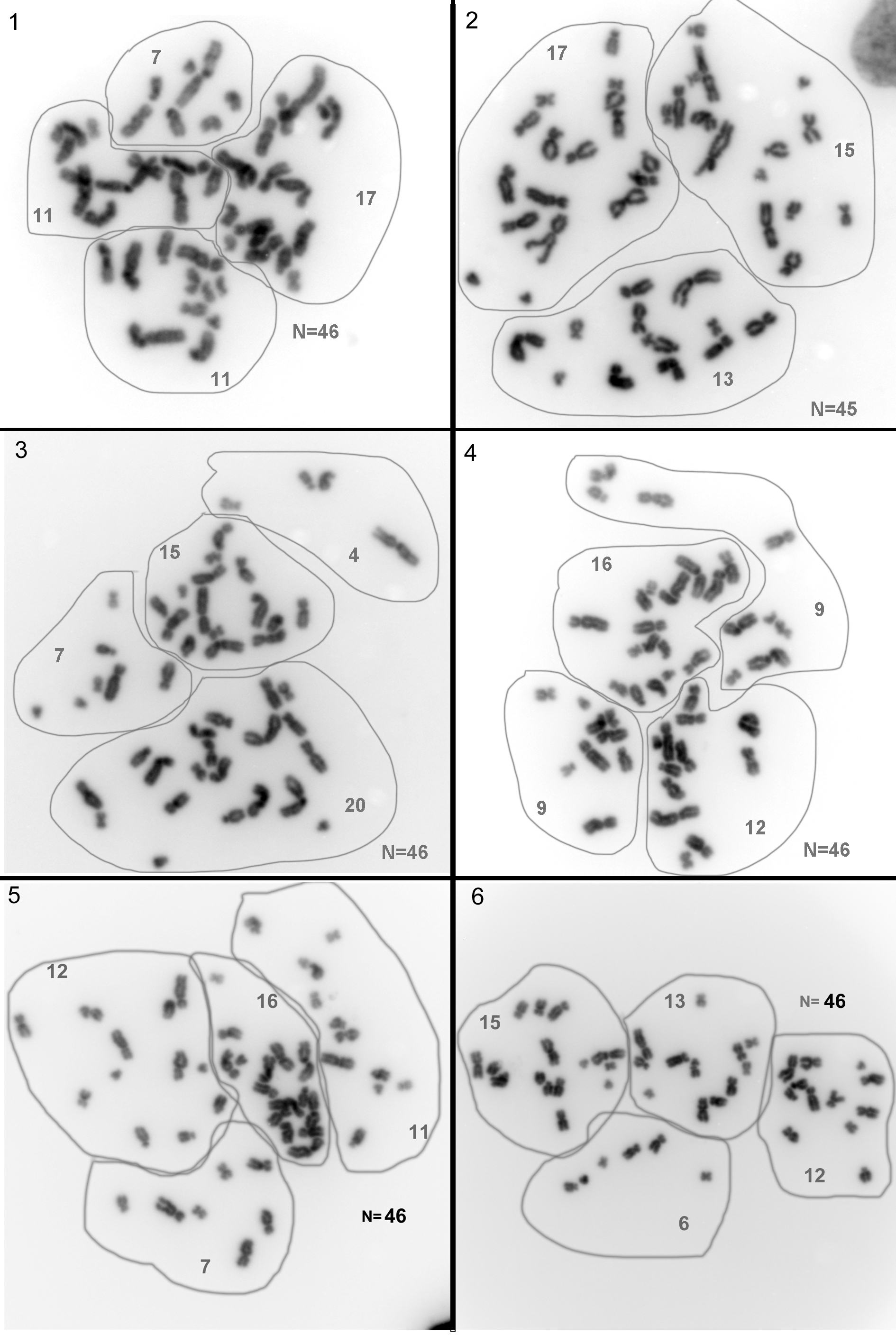

Supplement: Supplementary file 3 — Additional file 3. Metaphase spreads of BJ1 cells: Five out of six randomly selected Giemsa stained metaphase spreads reveal an inconspicuous diploid chromosome set of n = 46, XY (n = 45 in metaphase 2 is likely due to loss of one chromosome during preparation). [file 13072_2017_146_MOESM3_ESM.jpg]

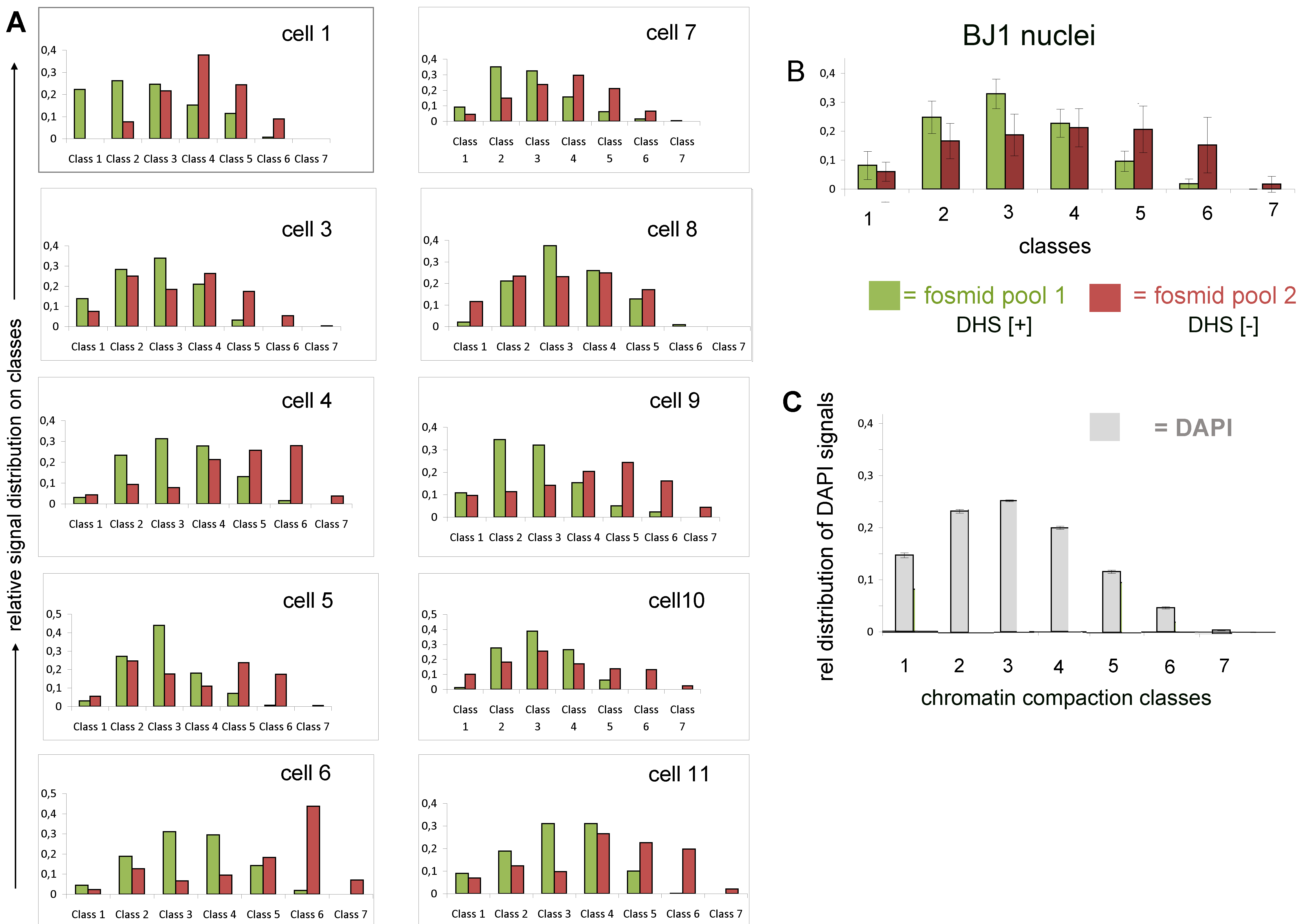

Supplement: Supplementary file 5 — Additional file 5. Single-cell profiles for target sites of fosmid pools 1 and 2 mapped to chromatin compaction classes in BJ1 cells for illustration of intercellular variability. (A) Mapping profiles from ten randomly chosen individual nuclei illustrate consistent distinct distribution profiles of fosmid pool 1 (green) toward low chromatin compaction classes and of pool 2 (red) toward higher chromatin compaction classes. (B) Standard deviations of relative probe signal distributions of all evaluated nuclei (compare Fig. 4 for standard errors of the mean (SEM). (C) Standard deviations of DAPI signal distribution on classes (compare Fig. 4 for SEM). [file 13072_2017_146_MOESM5_ESM.jpg]

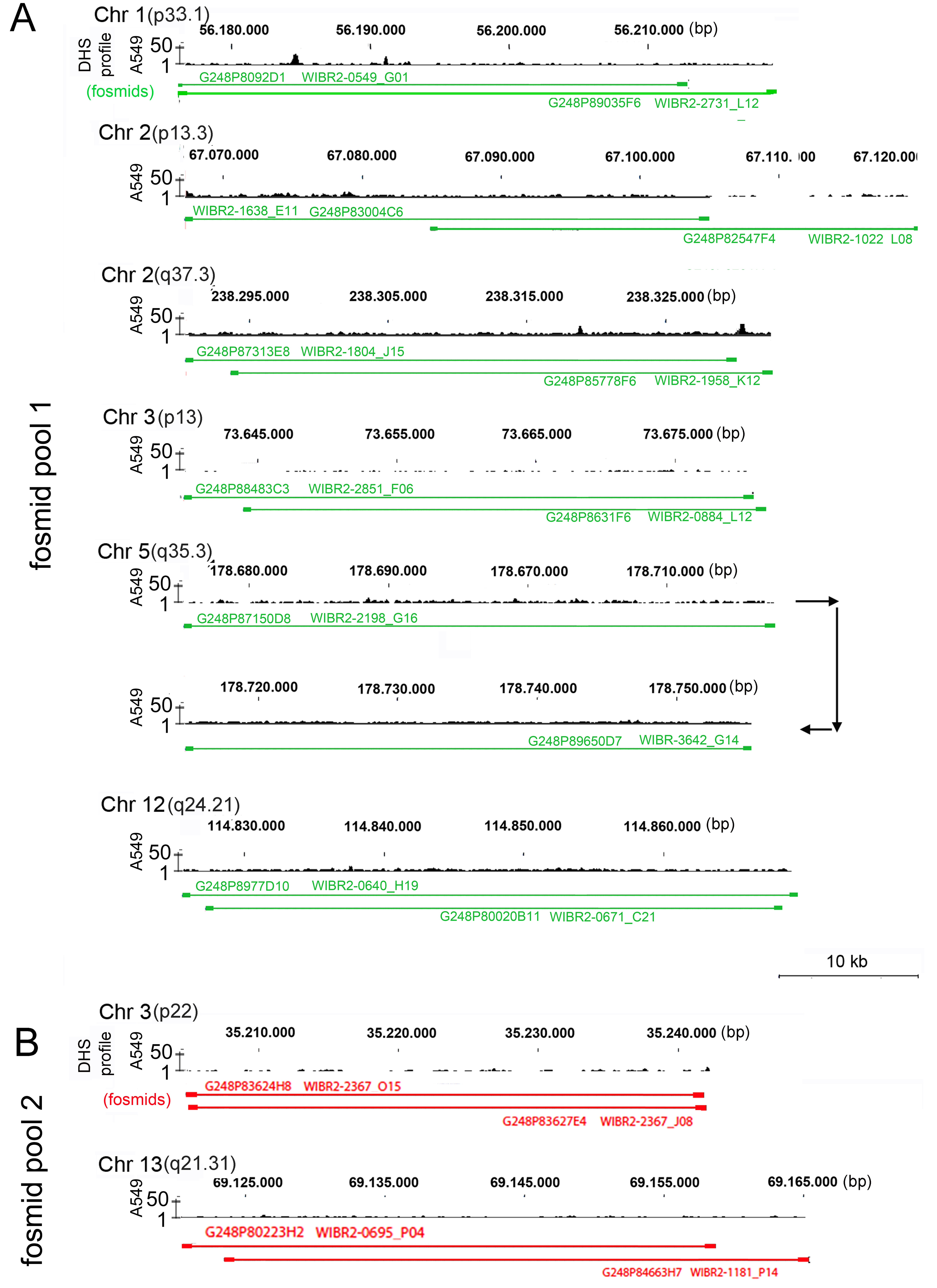

Supplement: Supplementary file 6 — Additional file 6. DHS profiles and fosmid clones used for target regions delineating DHS[−] sites on different chromosomes in A549 nuclei. (A) Selected regions with clones of fosmid pool 1 (green) and (B) with clones of fosmid pool 2 (red). DHS profile in black (browser shots adopted from http://encodeproject.org/). Note: probe sets are identical to probe sets shown in Fig. 3. [file 13072_2017_146_MOESM6_ESM.jpg]

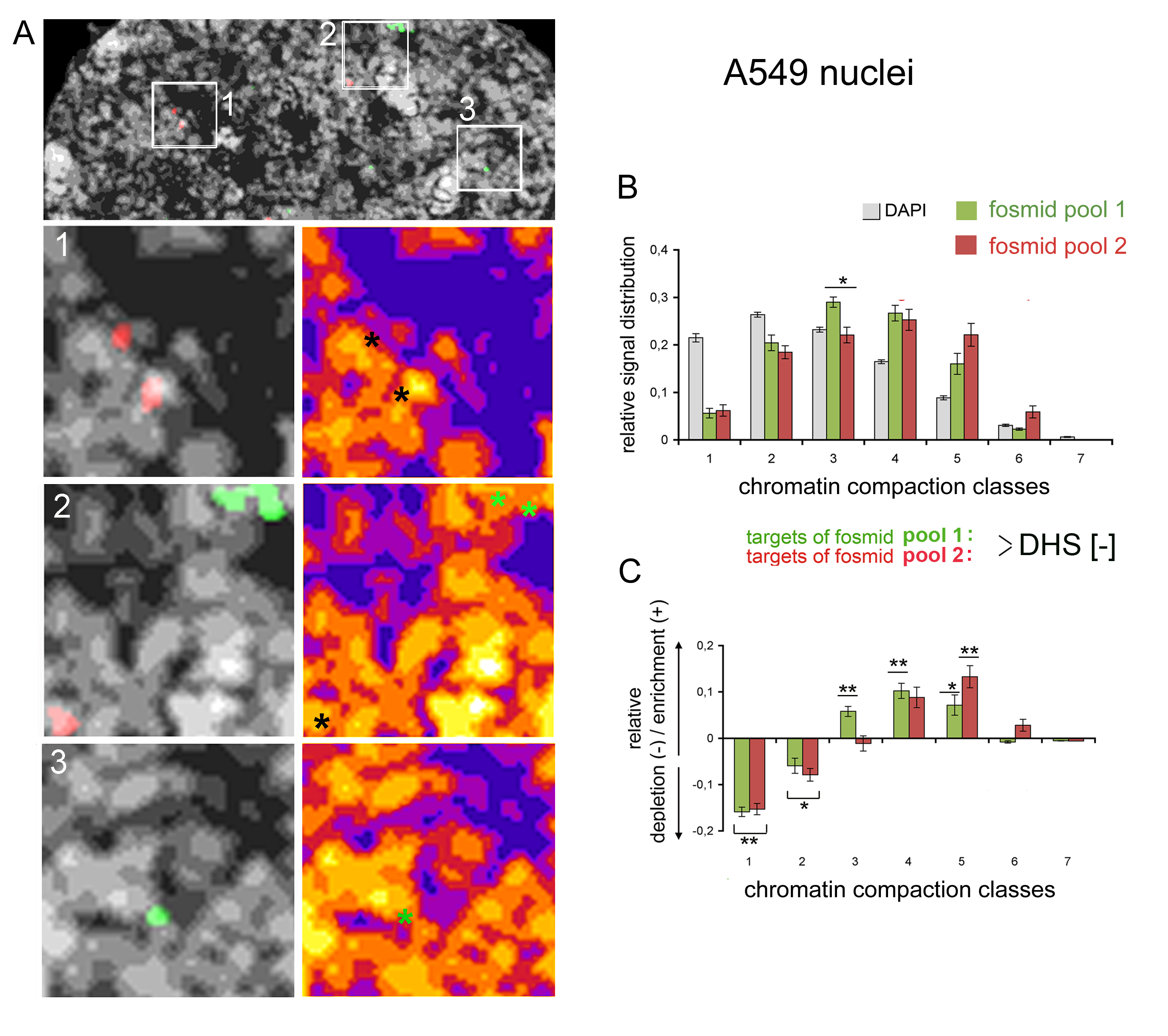

Supplement: Supplementary file 9 — Additional file 9. 3D nuclear topography and quantitative mapping of ~40 kb targets of DHS[−] regions in A549 nuclei. (A) Part of a SIM light-optical section from a whole nucleus acquisition with representative inset magnifications. DAPI-stained DNA after intensity classification shown as gray gradations and color heat map, respectively. Segmented signals delineating targets both of fosmid pool 1 (green) and fosmid pool 2 (red) show a similar location with regard to chromatin compaction classes (asterisks in color heat maps, pool 1 (green), pool 2 (black). Scale bar 2 µm, insets 0.5 µm. (B) Quantified distributions (N = 10 nuclei) of fosmid pools 1 (green) and 2 (red) within respective chromatin compaction classes (all classes shown in gray). (C) Quantified levels of relative enrichment (positive values) or depletion (negative values) of fosmid pool 1 and pool 2 signals show an enrichment of signals in higher compaction classes. Error bars = standard deviation of the mean *p ≤ 0.05, **p ≤ 0.01, ***p ≤ 0.001. [file 13072_2017_146_MOESM9_ESM.jpg]

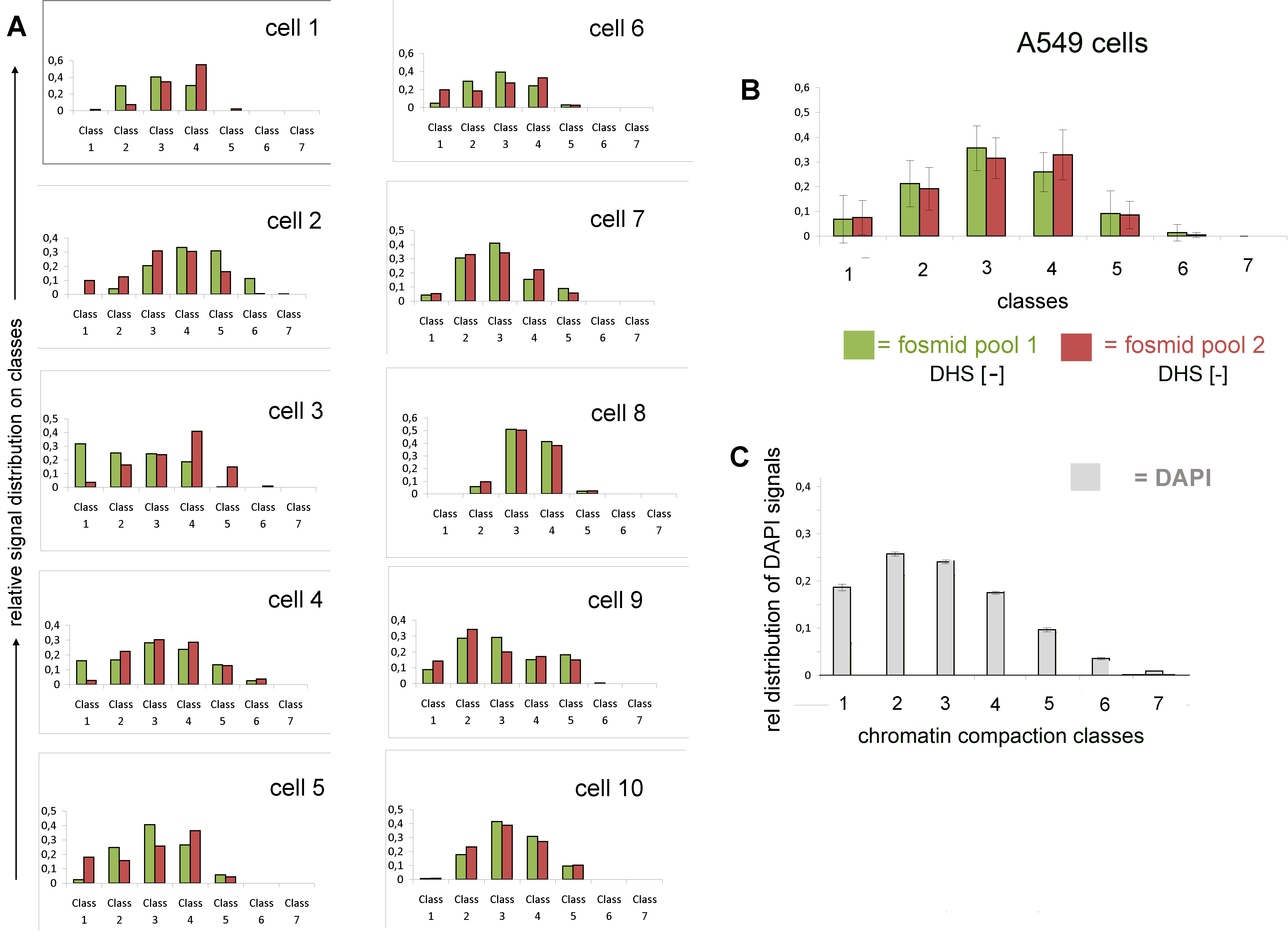

Supplement: Supplementary file 10 — Additional file 10. Single-cell profiles for target sites of fosmid pools 1 and 2 mapped to chromatin compaction classes in A549 cells for illustration of intercellular variability. (A) Mapping profiles of A549 nuclei (N = 10) illustrate for most nuclei fairly similar distribution profiles of fosmid pool 1 (green) and fosmid pool 2 (red). (B) Standard deviations of relative probe signal distributions of all evaluated nuclei (compare Additional file 8 for standard errors of the mean (SEM). (C) Standard deviations of DAPI signal distribution on classes (compare Additional file 8 for SEM). [file 13072_2017_146_MOESM10_ESM.jpg]

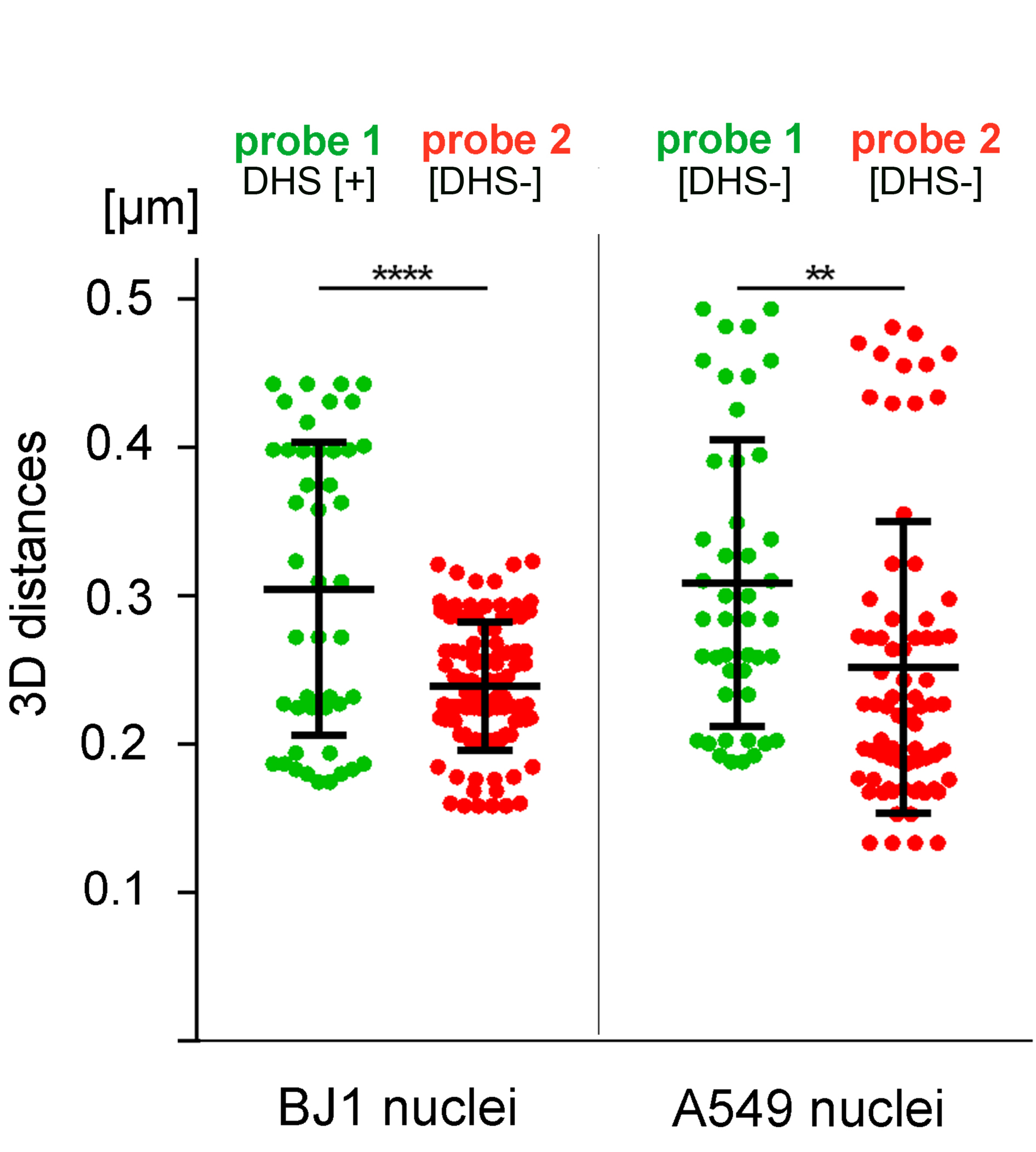

Supplement: Supplementary file 16 — Additional file 16. 3D distances between centroids of 6-kb probe 1 (green) and centroids of 6-kb probe 2 in BJ1 (left) and in A549 cells (right). Distance measurements are restricted to distances <500 nm presumably comprising only sister chromatids of S/G2 nuclei. The smaller distances between centroids of green signals (probe 1; DHS[+] in BJ1 cells, DHS[−] in A549 cells) compared to distances between centroids of red signals (probe 2; DHS[−] both in BJ1 and A549 cells) hint to a consistent orientation of these segments irrespective of DNAse I sensitivity. [file 13072_2017_146_MOESM16_ESM.jpg]

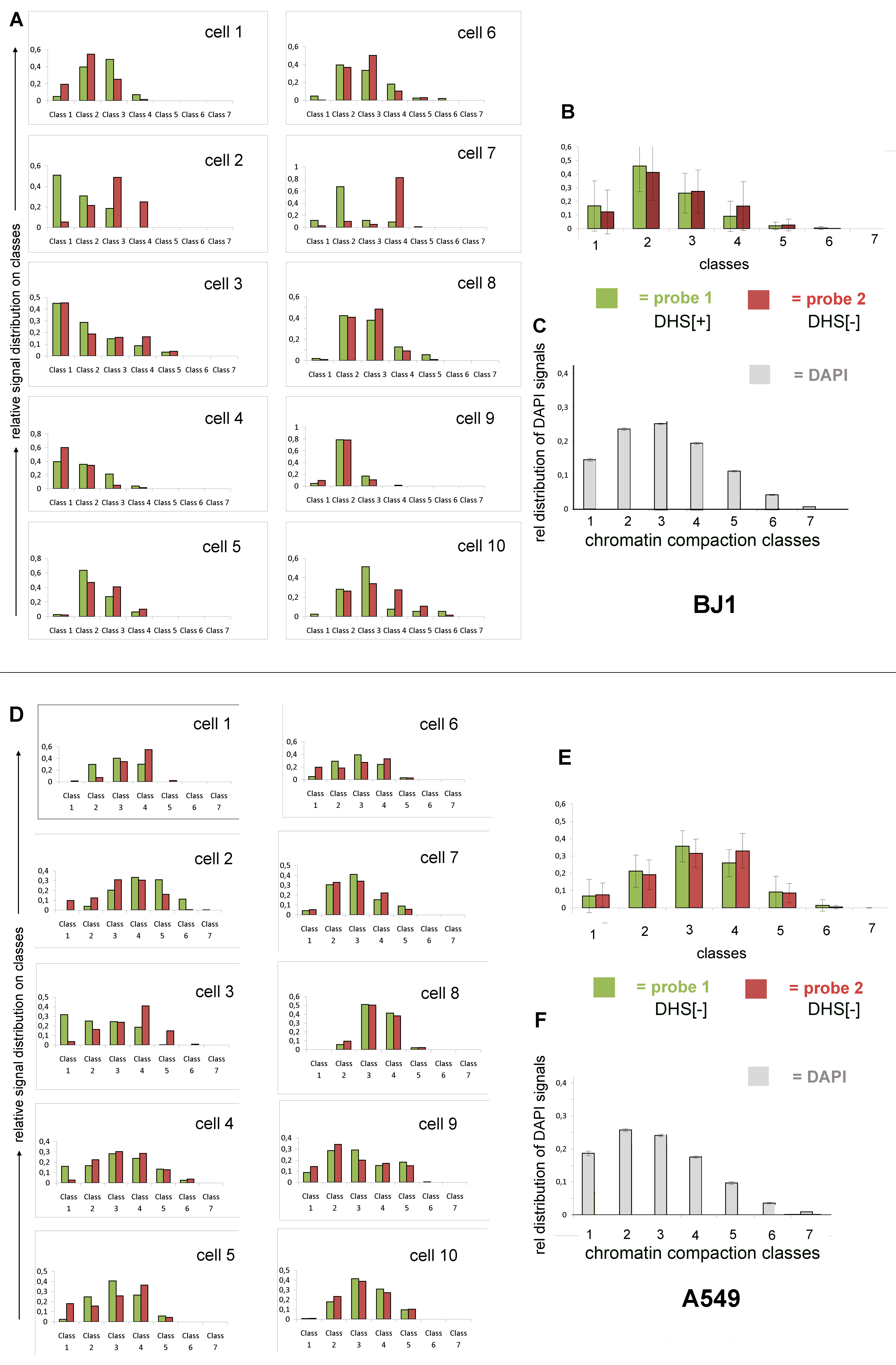

Supplement: Supplementary file 17 — Additional file 17. Single-cell profiles of 6-kb probes 1 and 2 targets mapped to chromatin compaction classes in BJ1 (A–C) and A549 cells (D–F) for illustration of intercellular variability. (A) Mapping profiles from ten randomly chosen individual BJ1 nuclei for illustration of intercellular variabilities and similarities of relative signal distribution of probe 1 (green) and probe 2 (red) within DAPI intensity classes. Note an only marginal signal representation in classes 5–7. (B) Standard deviations of relative probe signal distributions of all evaluated nuclei (compare Fig. 6 for standard errors of the mean (SEM). (C) Standard deviations of DAPI signal distribution on classes (compare Fig. 6 for standard errors of the mean (SEM). (D) Respective mapping profiles from 10 individual A549 nuclei of relative signal distribution of probe 1 (green) and probe 2 (red) within DAPI intensity classes. Profiles show an overall broader distribution range compared to BJ1 nuclei. (E) Standard deviations of relative probe signal distributions of all evaluated nuclei (compare Fig. 6 for standard errors of the mean (SEM). (F) Standard deviations of DAPI signal distribution on classes (compare Fig. 6 for standard errors of the mean (SEM). [file 13072_2017_146_MOESM17_ESM.jpg]

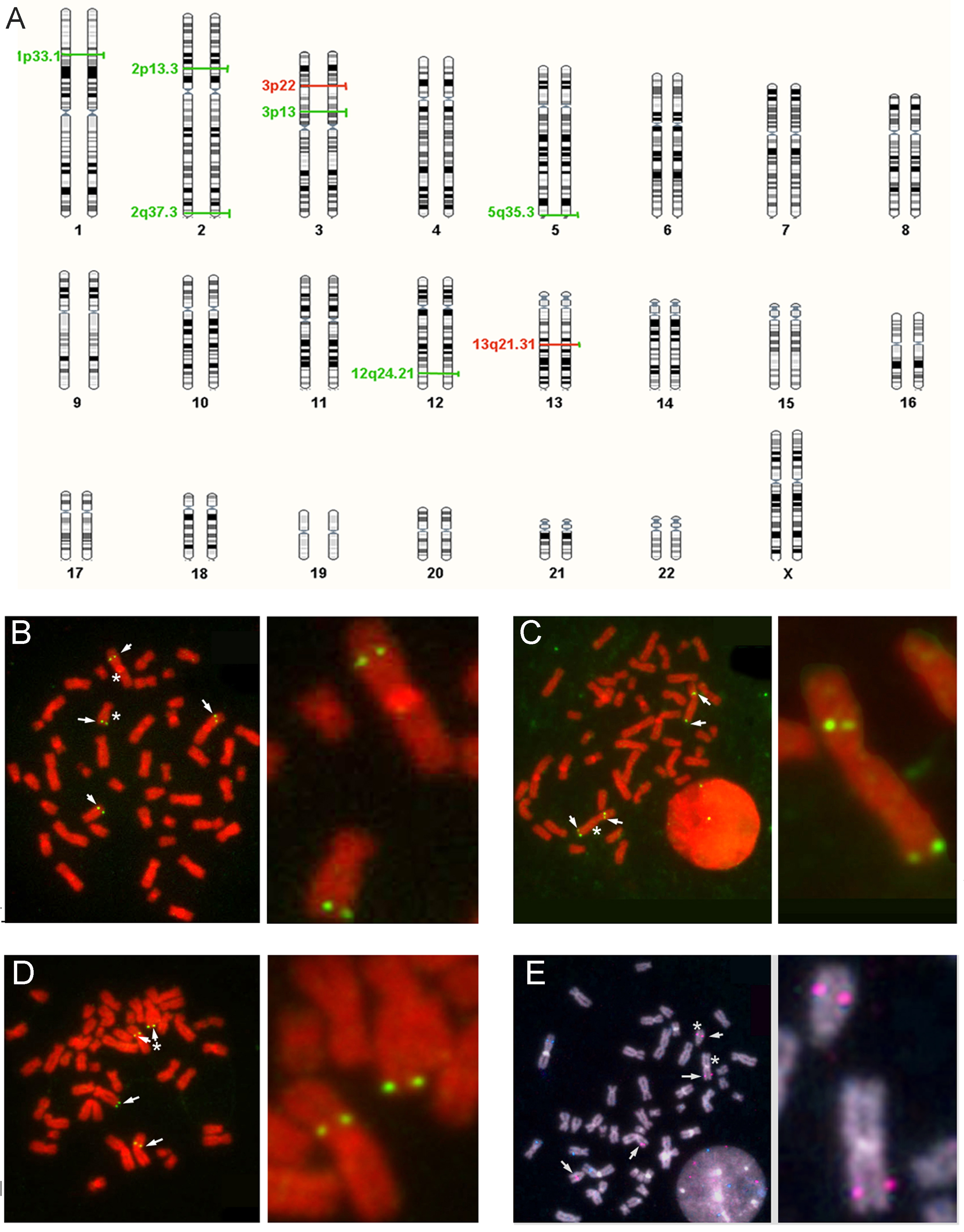

Supplement: Supplementary file 21 — Additional file 21. FISH of fosmid pairs on normal human metaphases for verification of specificity. (A) Human metaphase ideogram with marked positions of tested fosmids. (B–E) Metaphase spreads after FISH with (B) fosmid pairs G248P8092D1/G248P89035F6 mapped on 1p and G248P80020B1/G248P8977D10 mapped on 12q, (C) G248P83004C6/G248P82547F4 mapped on 2p and G248P87313E8/G248P85778F6 mapped on 2q, (D) G248P8631F6/G248P88483C3 mapped on 3p and G248P87150D8/G248P89650D7 mapped on 5q, (E) G248P83624H8/G248P83627E4 mapped on 3p and G248P80223H2/G248P84663H7 mapped on 13q. All tested probes show a specific hybridization signal at the expected chromosomal position. [file 13072_2017_146_MOESM21_ESM.jpg]
