# Supplementary material for: Initial high-resolution microscopic mapping of active and inactive regulatory sequences proves non-random 3D arrangements in chromatin domain clusters
Source: Epigenetics Chromatin. 2017 Aug 7;10:39. doi: 10.1186/s13072-017-0146-0 (PMC5547466; doi:10.1186/s13072-017-0146-0)
Supplement: Supplementary file 7 — Additional file 7. M-FISH karyotype analysis of A549 cells. (A) Representative karyotype obtained by M-FISH after combinatorial labeling of chromosome-specific paint probes with seven fluorochromes. (B) Quantitation of 20 metaphases reveals a karyotype with 62–66 chromosomes with consistent structural rearrangements involving chromosomes 1,2,3,6,8,11,15,19,20. This constellation allows for fosmid pool 1 up to 19, for fosmid pool 2 up to five distinct hybridization sites in a nucleus (compare Additional file 6). [file 13072_2017_146_MOESM7_ESM.pdf]

A

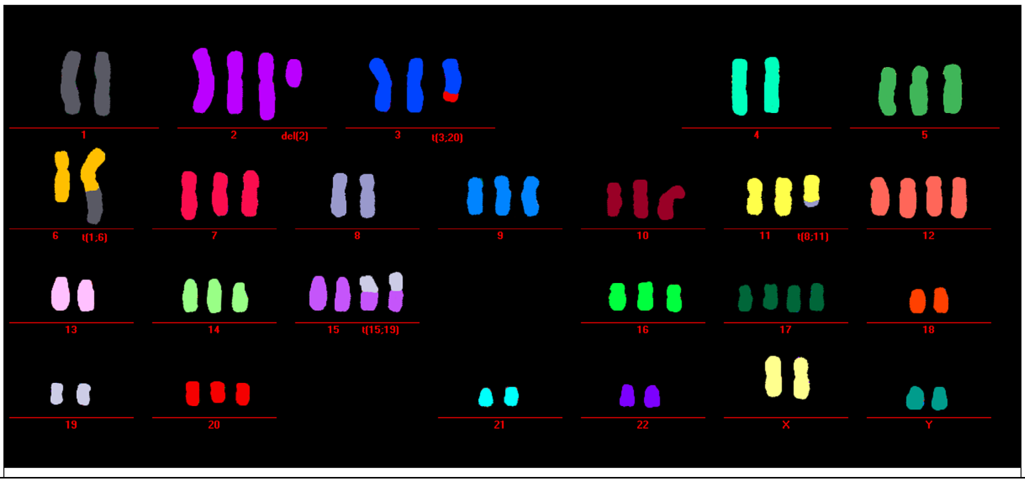

M-FISH: A549 adenocarcinoma cell line

B

Chromosome	Image 1 (n=64)	Image 2 (n=63)	Image 3 (n=64)	Image 4 (n=64)
**1**	1 x2	1 x2	1 x2	1 x2
**2**	2 x3<br>**del(2q)** x1	2 x3<br>**del(2q)** x1	2 x3<br>**del(2q)** x1	2 x3<br>**del(2q)** x1
**3**	3 x2<br>**t(3; 20)** x1	3 x2<br>**t(3; 20)** x1	3 x2<br>**t(3; 20)** x1	3 x2<br>**t(3; 20)** x1
**4**	4 x2	4 x2	4 x2	4 x2
**5**	5 x3	5 x3	5 x3	5 x3
**6**	6 x1<br>**t(1;6)** x1	6 x1<br>**t(1;6)** x1	6 x1<br>**t(1;6)** x1	6 x1<br>**t(1;6)** x1
**7**	7 x3	7 x3	7 x3	7 x3
**8**	8 x3	8 x3	8 x3	8 x3
**9**	9 x3	9 x3	9 x3	9 x3
**10**	10 x3	10 x3	10 x3	10 x3
**11**	11 x2<br>**t(8;11)** x1	11 x2<br>**t(8;11)** x1	11 x2<br>**t(8;11)** x1	11 x2<br>**t(8;11)** x1
**12**	12 x4	12 x3	12 x4	12 x4
**13**	13 x2	13 x2	13 x2	13 x2
**14**	14 x3	14 x3	14 x3	14 x3
**15**	15 x2<br>**t(15;19)** x2	15 x2<br>**t(15;19)** x2	15 x2<br>**t(15;19)** x2	15 x2<br>**t(15;19)** x2
**16**	16 x3	16 x3	16 x3	16 x3
**17**	17 x4	17 x4	17 x4	17 x4
**18**	18 x2	18 x2	18 x2	18 x2
**19**	19 x2	19 x2	19 x2	19 x2
**20**	20 x2	20 x3	20 x3	20 x2
**21**	21 x2	21 x2	21 x2	21 x2
**22**	22 x1	22 x2	22 x2	22 x2
**X**	X x2	X x1	X x2	X x2
**Y**	Y x1	Y x1	-	Y x1

Chromosome	Image 5 (n=62)	Image 6 (n=63)	Image 7 (n=63)	Image 8 (n=63)
**1**	1 x2	1 x2	1 x2	1 x2
**2**	2 x3<br>**del(2q)** x1	2 x3<br>**del(2q)** x1	2 x3<br>**del(2q)** x1	2 x3<br>**del(2q)** x1
**3**	3 x2<br>**t(3;20)** x1	3 x2<br>**t(3;20)** x1	3 x2<br>**t(3;20)** x1	3 x2<br>**t(3;20)** x1
**4**	4 x2	4 x2	4 x2	4 x2
**5**	5 x3	5 x3	5 x3	5 x3
**6**	6 x1<br>**-**	6 x1<br>**t(1;6) x1**	6 x1<br>**t(1;6) x1**	6 x1<br>**t(1;6) x1**
**7**	7 x3	7 x3	7 x3	7 x2
**8**	8 x3	8 x3	8 x3	8 x3
**9**	9 x3	9 x3	9 x3	9 x2<br>del(9q) x1
**10**	10 x3	10 x3	10 x3	10 x3
**11**	11 x2<br>**t(8;11)** x1	11 x2<br>**t(8;11)** x1	11 x2<br>**t(8;11)** x1	11 x2<br>**t(8;11)** x2
**12**	12 x2	12 x3	12 x3	12 x3
**13**	13 x2	13 x2	13 x2	13 x2
**14**	14 x3	14 x3	14 x3	14 x3
**15**	15 x2<br>**t(15;19)** x2	15 x2<br>**t(15;19)** x2	15 x2<br>**t(15;19)** x2	15 x2<br>**t(15;19)** x2
**16**	16 x2	16 x3	16 x3	16 x3
**17**	17 x4	17 x4	17 x4	17 x4
**18**	18 x2	18 x2	18 x2	18 x2
**19**	19 x2	19 x1	19 x2	19 x1
**20**	20 x3	20 x3	20 x3	20 x3
**21**	21 x2	21 x2	21 x2	21 x2
**22**	22 x2	22 x2	22 x2	22 x2
**X**	X x2	X x2	X x1	X x2
**Y**	Y x2	Y x1	Y x1	Y x1

**Chromosome**	**Image 9 (n=66)**	**Image 10 (n=66)**	**Image 11 (n=65)**	**Image 12 (n=66)**
**1**	1 x2	1 x2	1 x2	1 x2
**2**	2 x3<br>**del(2q)** x1	2 x3<br>**del(2q)** x1	2 x3<br>**del(2q)** x1	2 x3<br>**del(2q)** x1
**3**	3 x2<br>**t(3;20)** x1	3 x2<br>**t(3;20)** x1	3 x2<br>**t(3;20)** x1	3 x2<br>**t(3;20)** x1
**4**	4 x2	4 x2	4 x2	4 x1<br>t(Y;4) x1
**5**	5 x3	5 x3	5 x3	5 x3
**6**	6 x1<br>**t(1;6) x1**	6 x1<br>**t(1;6) x1**	6 x1<br>**t(1;6) x1**	6 x1<br>**t(1;6) x1**
**7**	7 x3	7 x3	7 x3	7 x3
**8**	8 x3	8 x3	8 x3	8 x3
**9**	9 x3	9 x3	9 x3	9 x3
**10**	10 x3	10 x3	10 x3	10 x3
**11**	11 x2<br>**t(8;11)** x1	11 x2<br>**t(8;11)** x1	11 x2<br>**t(8;11)** x1	11 x2<br>**t(8;11)** x1
**12**	12 x4	12 x4	12 x4	12 x4
**13**	13 x2	13 x2	13 x2	13 x2
**14**	14 x3	14 x3	14 x3	14 x3
**15**	15 x2<br>**t(15;19)** x2	15 x2<br>**t(15;19)** x2	15 x2<br>**t(15;19)** x2	15 x2<br>**t(15;19)** x2
**16**	16 x3	16 x3	16 x3	16 x3
**17**	17 x4	17 x4	17 x4	17 x4
**18**	18 x2	18 x2	18 x2	18 x2
**19**	19 x2	19 x2	19 x2	19 x2
**20**	20 x3	20 x3	20 x3	20 x3
**21**	21 x2	21 x2	21 x1	21 x2
**22**	22 x2	22 x2	22 x2	22 x2
**X**	X x2	X x2	X x2	X x2
**Y**	Y x2	Y x2	Y x2	Y x1<br>del(Yq) x1

Chromosome	Image 13<br>(n=64)	Image 14<br>(n=66)	Image 15<br>(n=65)	Image 16<br>(n=66)
**1**	1 x2	1 x2	1 x2	1 x2
**2**	2 x3<br>**del(2q)** x1	2 x3<br>**del(2q)** x1	2 x3<br>**del(2q)** x1	2 x4<br>**del(2q)** x1
**3**	3 x2<br>**t(3;20)** x1	3 x2<br>**t(3;20)** x1	3 x2<br>**t(3;20)** x1	3 x2<br>**t(3;20)** x1
**4**	4 x2	4 x2	4 x2	4 x2
**5**	5 x3	5 x3	5 x3	5 x3
**6**	6 x1<br>**t(1;6) x1**	6 x2<br>**-**	6 x1<br>**t(1;6) x1**	6 x1<br>**t(1;6) x1**
**7**	7 x3	7 x3	7 x3	7 x3
**8**	8 x3	8 x3	8 x3	8 x3
**9**	9 x3	9 x3	9 x3	9 x3
**10**	10 x3	10 x3	10 x3	10 x3
**11**	11 x2<br>**t(8;11)** x1	11 x2<br>**t(8;11)** x1	11 x2<br>**t(8;11)** x1	11 x2<br>**t(8;11)** x1
**12**	12 x3	12 x4	12 x4	12 x4
**13**	13 x2	13 x2	13 x2	13 x2
**14**	14 x3	14 x3	14 x3	14 x3
**15**	15 x2<br>**t(15;19)** x2	15 x2<br>**t(15;19)** x2	15 x2<br>**t(15;19)** x2	15 x2<br>**t(15;19)** x2
**16**	16 x3	16 x3	16 x3	16 x3
**17**	17 x4	17 x4	17 x4	17 x4
**18**	18 x2	18 x2	18 x2	18 x2
**19**	19 x2	19 x2	19 x2	19 x2
**20**	20 x3	20 x3	20 x3	20 x3
**21**	21 x2	21 x2	21 x2	21 x2
**22**	22 x2	22 x2	22 x2	22 x2
**X**	X x2	X x2	X x2	X x2
**Y**	Y x1	Y x2	Y x1	Y x2

Chromosome	Image 17 (n=65)	Image 18 (n=65)	Image 19 (n=65)	Image 20 (n=66)
**1**	1 x2	1 x2	1 x2	1 x2
**2**	2 x3<br>**del(2q)** x1	2 x3<br>**del(2q)** x1	2 x3<br>**del(2q)** x1	2 x3<br>**del(2q)** x1
**3**	3 x2<br>**t(3;20)** x1	3 x2<br>**t(3;20)** x1	3 x2<br>**t(3;20)** x1	3 x2<br>**t(3;20)** x1
**4**	4 x2	4 x2	4 x2	4 x2
**5**	5 x3	5 x3	5 x3	5 x3
**6**	6 x1<br>**t(1;6) x1**	6 x1<br>**t(1;6) x1**	6 x1<br>**t(1;6) x1**	6 x1<br>**t(1;6) x1**
**7**	7 x3	7 x3	7 x3	7 x3
**8**	8 x3	8 x3	8 x3	8 x3
**9**	9 x3	9 x3	9 x3	9 x3
**10**	10 x3	10 x3	10 x3	10 x3
**11**	11 x2<br>**t(8;11)** x1	11 x2<br>**t(8;11)** x1	11 x2<br>**t(8;11)** x1	11 x2<br>**t(8;11)** x1
**12**	12 x3	12 x3	12 x4	12 x4
**13**	13 x2	13 x2	13 x2	13 x2
**14**	14 x3	14 x3	14 x3	14 x3
**15**	15 x2<br>**t(15;19)** x2	15 x2<br>**t(15;19)** x2	15 x2<br>**t(15;19)** x2	15 x2<br>**t(15;19)** x2
**16**	16 x3	16 x3	16 x3	16 x3
**17**	17 x4	17 x4	17 x4	17 x4
**18**	18 x2	18 x2	18 x2	18 x2
**19**	19 x2	19 x2	19 x2	19 x2
**20**	20 x3	20 x3	20 x3	20 x3
**21**	21 x2	21 x2	21 x2	21 x2
**22**	22 x2	22 x2	22 x2	22 x2
**X**	X x2	X x2	X x2	X x2
**Y**	Y x2	Y x2	Y x1	Y x2
