# Supplementary material for: Initial high-resolution microscopic mapping of active and inactive regulatory sequences proves non-random 3D arrangements in chromatin domain clusters
Source: Epigenetics Chromatin. 2017 Aug 7;10:39. doi: 10.1186/s13072-017-0146-0 (PMC5547466; doi:10.1186/s13072-017-0146-0)
Supplement: Supplementary file 11 — Additional file 11. Significance values for relative signal distributions of fosmid pools 1 and 2 in BJ1 and A549 cells. [file 13072_2017_146_MOESM11_ESM.pdf]

| | | | | |
|---|---|---|---|---|
| **BJ1** | **DAPI vs fosmid pool 1 [DHS+]**<br>class 1 : <0.001<br>class 2 : 0.468<br>class 3 : <0.001<br>class 4 : 0.062 (just above border)<br>class 5 : 0.027<br>class 6 : <0.001<br>class 7 : <0.001<br><br>**DAPI vs fosmid pool 2 [DHS-]**<br>class 1 : <0.001<br>class 2 : <0.001<br>class 3 : <0.001<br>class 4 : 0.455<br>class 5 : <0.001<br>class 6 : <0.001<br>class 7 : 0.168<br><br>**fosmid pool 1 [DHS+] vs fosmid pool 2 [DHS-]**<br>class 1 : 0.136<br>class 2 : <0.001<br>class 3 : <0.001<br>class 4 : 0.506<br>class 5 : <0.001<br>class 6 : <0.001<br>class 7 : 0.001 | | **A549** | **DAPI vs fosmid pool 1 [DHS-]**<br>class 1 : 0.008<br>class 2 : 0.032<br>class 3 : 0.008<br>class 4 : 0.008<br>class 5 : 0.032<br>class 6 : 0.690<br>class 7 : 0.018<br><br>**DAPI vs fosmid pool 2 [DHS-]**<br>class 1 : 0.008<br>class 2 : 0.056<br>class 3 : 0.310<br>class 4 : 0.151<br>class 5 : 0.008<br>class 6 : 0.421<br>class 7 : 0.007<br><br>**fosmid pool 1 [DHS-] vs fosmid pool 2 [DHS-]**<br>class 1 : 0.841<br>class 2 : 0.841<br>class 3 : 0.032<br>class 4 : 0.690<br>class 5 : 0.222<br>class 6 : 0.310<br>class 7 : 0.424 |
