# Supplementary material for: Initial high-resolution microscopic mapping of active and inactive regulatory sequences proves non-random 3D arrangements in chromatin domain clusters
Source: Epigenetics Chromatin. 2017 Aug 7;10:39. doi: 10.1186/s13072-017-0146-0 (PMC5547466; doi:10.1186/s13072-017-0146-0)
Supplement: Supplementary file 18 — Additional file 18. Significance values for relative signal distributions of 6-kb probes 1 and 2 in BJ1 and A549 nuclei. [file 13072_2017_146_MOESM18_ESM.pdf]

**BJ1 6kb probes 1 and 2**	**DAPI vs probe1 [DHS+]**<br>class 1 : 0.164 (hints to extension into class1)<br>class 2 : <0.001<br>class 3 : 0.989<br>class 4 : <0.001<br>class 5 : <0.001<br>class 6 : <0.001<br>class 7 : <0.001<br><br>**DAPI vs probe 2 [DHS-]**<br>class 1 : 0.017 (less extension into class 1)<br>class 2 : 0.001<br>class 3 : 0.989<br>class 4 : 0.010<br>class 5 : <0.001<br>class 6 : <0.001<br>class 7 : <0.001<br><br>**probe 1 [DHS+] vs probe 2 [DHS-]**<br>class 1 : 0.490<br>class 2 : 0.413<br>class 3 : 1.000<br>class 4 : 0.059<br>class 5 : 0.988<br>class 6 : 0.299<br>class 7 : NA (not represented in class 7)		**A549 6kb probes 1 and 2**	**DAPI vs probe1 [DHS-]**<br>class 1 : 0.005<br>class 2 : 0.796<br>class 3 : 0.007<br>class 4 : 0.035<br>class 5 : 0.393<br>class 6 : 0.003<br>class 7 : <0.001<br><br>**DAPI vs probe 2 [DHS-]**<br>class 1 : 0.002<br>class 2 : 0.023<br>class 3 : 0.011<br>class 4 : <0.001<br>class 5 : 0.853<br>class 6 : <0.001<br>class 7 : <0.001<br><br>**probe 1 [DHS-]vs probe 2 [DHS-]**<br>class 1 : 0.448<br>class 2 : 0.579<br>class 3 : 0.247<br>class 4 : 0.165<br>class 5 : 0.739<br>class 6 : 0.825<br>class 7 : 0.368
