# Supplementary material for: Initial high-resolution microscopic mapping of active and inactive regulatory sequences proves non-random 3D arrangements in chromatin domain clusters
Source: Epigenetics Chromatin. 2017 Aug 7;10:39. doi: 10.1186/s13072-017-0146-0 (PMC5547466; doi:10.1186/s13072-017-0146-0)
Supplement: Supplementary file 19 — Additional file 19. fosmid ID (G248 library #) and sequence alignment of fosmids used. Data are based on hg19. [file 13072_2017_146_MOESM19_ESM.pdf]

Fosmid ID (G248 library #)	Chr. position	Start (bp)	End (bp)	Length (kb)	full length of pair (kb)
G248P8092D1	1p33.1	56173094	56212987	39.9	46.0
G248P89035F6	1.p33.1	56173094	56219121	42.9	
G248P83004C6	2p13.3	67067250	67104909	37.7	52.7
G248P82547F4	2p13.3	67084911	67120006	35.1	
G248P87313E8	2q37.3	238290483	238330135	39.7	42
G248P85778F6	2q37.3	238293690	238332485	38.8	
G248P8631F6	3p13	73639648	73680498	40.8	43.3
G248P88483C3	3p13	73643931	73682963	39.0	
G248P87150D8	5q35.3	178675290	178717726	42.4	80
G248P89650D7	5q35.3	178714697	178755396	40.7	
G248P80020B11	12q24.21	114827219	114868439	41.2	43.6
G248P8977D10	12q24.21	114825587	114869204	43.6	
G248P83624H8	3p22	35204496	35241461	36.7	37.5
G248P83627E4	3p22	35204498	35241974	37.5	
G248P80223H2	13q21.31	69120342	69158436	38.1	44.8
G248P84663H7	13q21.31	69123320	69165157	41.8	
